# Chromatin-mediated feed-forward auxin biosynthesis in floral meristem determinacy

**DOI:** 10.1038/s41467-018-07763-0

**Published:** 2018-12-11

**Authors:** Nobutoshi Yamaguchi, Jiangbo Huang, Yoshitaka Tatsumi, Masato Abe, Shigeo S. Sugano, Mikiko Kojima, Yumiko Takebayashi, Takatoshi Kiba, Ryusuke Yokoyama, Kazuhiko Nishitani, Hitoshi Sakakibara, Toshiro Ito

**Affiliations:** 1 Division of Biological Science, Graduate School of Science and Technology, Nara Institute of Science and Technology, 8916-5, Takayama, Ikoma, Nara 630-0192 Japan; 2 Precursory Research for Embryonic Science and Technology, Japan Science and Technology Agency, 4-1-8, Honcho, Kawaguchi-shi, Saitama 332-0012 Japan; 3 Temasek Life Sciences Laboratory, 1 Research Link, National University of Singapore, Singapore, 117604 Republic of Singapore; 4 Ritsumeikan Global Innovation Research Organization, Ritsumeikan University, 1-1-1, Shiga, 525-8577 Japan; 5 RIKEN Center for Sustainable Resource Science, 1-7-22, Suehiro, Tsurumi, Yokohama 230-0045 Japan; 6 Department of Applied Biosciences, Graduate School of Bioagricultural Sciences, Nagoya University, Chikusa, Nagoya 464-8601 Japan; 7 Graduate School of Life Sciences, Tohoku University, Aoba-ku, Sendai 980-8578 Japan

## Abstract

In flowering plants, the switch from floral stem cell maintenance to gynoecium (female structure) formation is a critical developmental transition for reproductive success. In *Arabidopsis thaliana*, AGAMOUS (AG) terminates floral stem cell activities to trigger this transition. Although *CRABS CLAW* (*CRC*) is a direct target of AG, previous research has not identified any common targets. Here, we identify an auxin synthesis gene, *YUCCA4* (*YUC4*) as a common direct target. Ectopic *YUC4* expression partially rescues the indeterminate phenotype and cell wall defects that are caused by the *crc* mutation. The feed-forward *YUC4* activation by AG and CRC directs a precise change in chromatin state for the shift from floral stem cell maintenance to gynoecium formation. We also showed that two auxin-related direct CRC targets, *YUC4* and *TORNADO2*, cooperatively contribute to the termination of floral stem cell maintenance. This finding provides new insight into the CRC-mediated auxin homeostasis regulation for proper gynoecium formation.

## Abstract (web summary)

In *Arabidopsis*, the AG and CRC transcription factors terminate floral stem cells and allow the emergence of female floral organs. Here the authors show that AG and CRC form a feed-forward loop that controls local auxin biosynthesis via induction of *YUCCA4* to ensure successful gynoecium formation.

## Introduction

Flowers are reproductive organs in angiosperms^1^. Each flower is composed of four different types of floral organs: sepals, petals, stamens, and carpel^1^. These floral organs are generated from floral meristems, which each contain a small pool of pluripotent and undifferentiated stem cells^2^. To form the appropriate number of floral organs, the rate of stem cell proliferation must be coordinated with the rate of organ formation. Such balances are determined by floral meristem termination (also known as floral determinacy). If floral meristems terminate too early, the cells are unable to produce the precise number of floral organs. In contrast, delayed or no floral meristem termination leads to failure in reproductive organ and sterility. Thus, floral meristem termination is critical for allocating resources for the next generation.

The MADS-box transcription factor AGAMOUS (AG) is considered to be a master regulator of floral meristem termination in *Arabidopsis*^3^. A loss of AG function results in indeterminate growth of floral meristems^3–5^. Interplay between AG and APETALA2 (AP2) regulates the expression of hundreds of targets genes^6^. Previous studies have only identified a handful of direct AG targets that control determinacy. Such targets include the homeodomain transcription factor WUSCHEL (WUS)^7,8^, the C2H2 zinc-finger protein KNUCKLES (KNU)^9–11^, and the YABBY transcription factor CRABS CLAW (CRC)^12–17^. WUS has a central role in floral meristem determinacy. AG binds directly to nucleotide sequences near the *WUS* transcription start site and in its 3′ UTR to repress its expression^8^. AG also binds to the promoter of a *WUS* repressor, *KNU*, at the site where a CArG box and a Polycomb response element (PRE) overlap^10,11^. Polycomb group (PcG) proteins bind to the PRE to deposit/maintain histone 3 lysine 27 (H3K27) trimethylation to silence transcription^18,19^. Eviction of the PcG proteins by AG triggers *KNU* expression in a cell division-dependent manner and hence suppresses *WUS* expression^11^. *CRC* expression is also directly controlled by AG^14,15,17^. CRC represses *TORNADO2* (*TRN2*) via an evolutionarily conserved and biologically functional *cis*-regulatory motif^17,20,21^. TRN2 modulates homeostasis of the morphogenic plant hormone auxin and contributes to *WUS* regulation^17^. Previous genetic evidence strongly suggests that an auxin-related target(s) acts downstream of CRC and in parallel with TRN2^17^. Auxin transport inhibitor treatment led to an almost full rescue of the *crc* mutant phenotype, while a *trn2* mutation led to a partial rescue^17,22^. Auxin homeostasis is controlled by multiple key regulators. For example, the two-step conversion of tryptophan to indole-3-acetic acid catalyzed by TRYPTOPHAN AMINOTRANSFERASE OF ARABIDOPSISs (TAAs) and YUCCAs (YUCs) is a critical step in the major pathway synthesizing bioactive auxin^23–25^. Auxin perception and signaling are controlled by auxin receptors, ubiquitin-ligase, and auxin-dependent transcriptional modules^26^. Despite their potential impact on auxin homeostasis, auxin-related CRC targets, other than *TRN2*, have not been identified.

After termination of floral meristems, rapid cell expansion and elongation direct organ growth and development. Auxin maxima in organ primordia correlate well with the maximal rates of primary cell wall expansion, which mainly requires cellulose, non-cellulosic polysaccharides, pectin, and glycoproteins^27–30^. Auxin results in the loosening and expansion of cell walls^31^, which requires interactions between multiple molecular factors to relax wall tension. Expansins are major regulators that mediate the local sliding of wall polymers by reducing adhesion between adjacent wall polysaccharides^32^. Xyloglucan endotransglucosylases/hydrolases catalyze cleavage of xyloglucan polymers and reconnect xyloglucan chains^33^. Although local differences in cell wall composition have an important role in meristem activity and its subsequent development, little is known about cell wall composition during floral meristem termination and their effects on stem cell activities.

In this study, we systematically identified genes that are transcriptionally regulated by both AG and CRC. We show that the auxin biosynthetic gene *YUC4* is a common direct target for both AG and CRC. AG and CRC synergistically regulate *YUC4* expression during floral meristem determinacy. Furthermore, we showed that two auxin-related CRC targets, *YUC4* and *TRN2* cooperatively function for floral meristem termination. Our results provide insight into the transcriptional cascades that govern the transition from floral stem cell maintenance to gynoecium formation.

## Results

### AG and CRC control cellular developmental processes

**Fig. 1:**
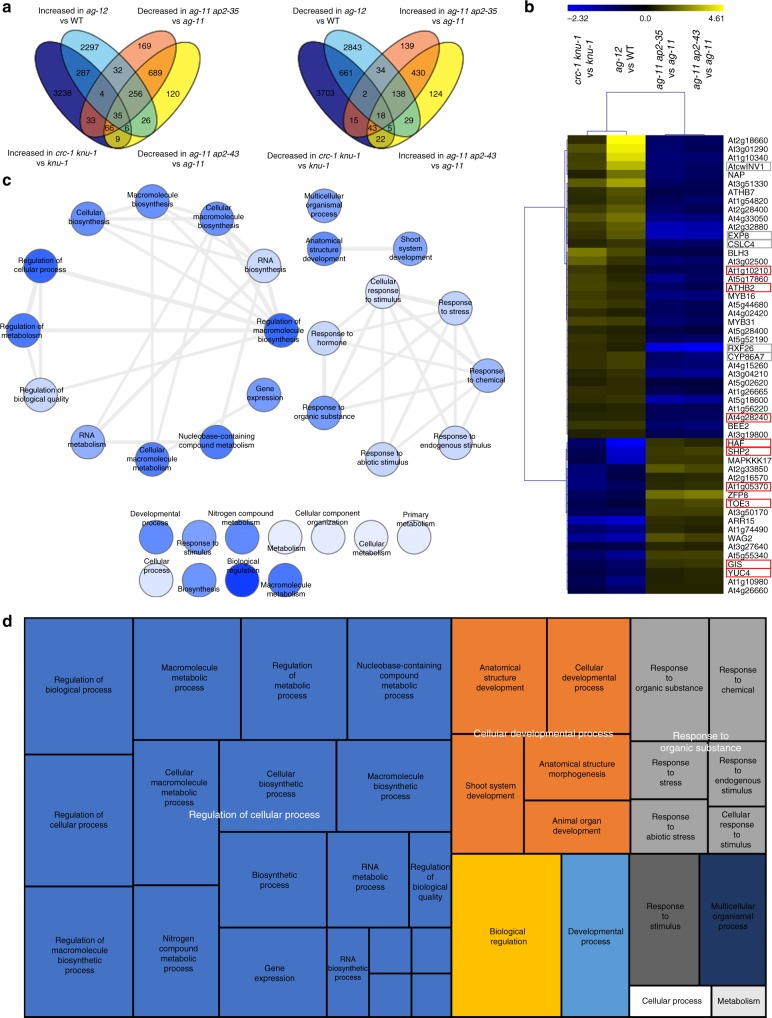
Identification of downstream targets of AG and CRC during flower development. **a** Venn diagrams showing the overlap between genes with increased/decreased expression in *crc-1 knu-1* vs *knu-1* and *ag-12* vs WT, and genes with decreased/increased expression in *ag-11 ap2-35* vs *ag-11* and *ag-11 ap2-43* vs *ag-11*. **b** Hierarchical clustering of 53 genes downstream of AG and CRC. The heat map displays the log_2_ fold change of differentially expressed genes based on public datasets. Red and gray boxes highlight AG direct targets and cell wall regulators, respectively. **c** Interactive graph view generated by REVIGO. Dark and light colors indicate lower and higher *p*-values, respectively. Circle sizes indicate the frequency of the GO term in the underlying GOA database. **d** TreeMap view of GO terms

To identify target genes that are regulated by both AG and CRC, we computationally reanalyzed four published transcriptome datasets^6,17,34^. First, we screened for genes that were differentially expressed in both *crc-1 knu-1* vs *knu-1*, and *ag-12* vs WT (Fig. 1a). This analysis identified 332 upregulated and 686 downregulated genes. Then, we examined how many of these genes were differentially expressed in opposite directions in the two *ap2* mutants^6^. Among the 1018 genes, 53 genes were restored to wild-type levels by introducing *ap2* mutations (Fig. 1a, b, Supplementary Fig. 1, and Supplementary Data 1).

To examine the potential functions of the 53 putative AG and CRC regulated genes, we performed a GO term enrichment analysis using agriGO and REVIGO^35^ (Supplementary Fig. 2 and Supplementary Data 2). Three major categories were visualized with an interactive graph view and TreeMap (Fig. 1c, d): Regulation of cellular processes, Cellular developmental processes, and Responses to organic substances. Regulation of cellular processes included GO terms related to transcription, such as ‘gene expression’, ‘RNA metabolism’, and ‘RNA biosynthesis’ (Fig. 1c, d). A cellular developmental process included the GO terms ‘cellular developmental process’, ‘anatomical structure morphology’, ‘anatomical structure development’, and ‘shoot system development’ (Fig. 1c, d). The GO term ‘anatomical structure process’ was associated with *YUC4*, and *EXPANSIN 8* (*EXP8*), which encoded an enzyme in the auxin biosynthesis pathway^25^ and an enzyme that alters cell wall polymers^36^, respectively. The Responses to organic substances category included various responses such as ‘response to chemical’, ‘cellular response to stimulus’, ‘response to stress’, ‘response to endogenous stimulus’, and ‘cellular response to stimulus’ (Fig. 1c, d). The genes associated with this category included *ARABISOPSIS THALIANA CELL WALL INVERTASE1* (*AtcwINV1*)^37^, *BR-ENHANCED EXPRESSION* (BEE2)^38^, and *ARABIDOPSIS RESPONSE REGULATOR15* (*ARR15*)^39^.

Then, we investigated how many of these 53 genes were bound by AG using a publicly available AG ChIP-seq dataset^40^. Among the 53 genes, 9 genes were significantly enriched by AG (Fig. 1b, red square). The 9 genes regulated by both AG and CRC were *MITOGEN-ACTIVATED PROTEIN KINASE 1* (*At1g10210*)^41^, *ARABIDOPSIS THALIANA HOMEOBOX PROTEIN 2* (*ATHB2*)^42^, wound-responsive family protein (*At4g28240*), *HALF FILLED* (*HAF*)^43^, *SHATTERPROOF 2* (*SHP2*)^44^, Sec14p-like phosphatidylinositol transfer family protein (*At1g05370*), *TARGET OF EAT 3* (*TOE3*)^45^, *GLABROUS INFLORESCENCE STEMS* (*GIS*)^46^, and *YUC4*^25^. Since a previous study proposed the existence of an auxin-related target (or targets) that act downstream of CRC and in parallel with TRN2^17^, we hypothesized that *YUC4* could be such a target.

### AG and CRC synergistically regulate *YUC4* expression

**Fig. 2:**
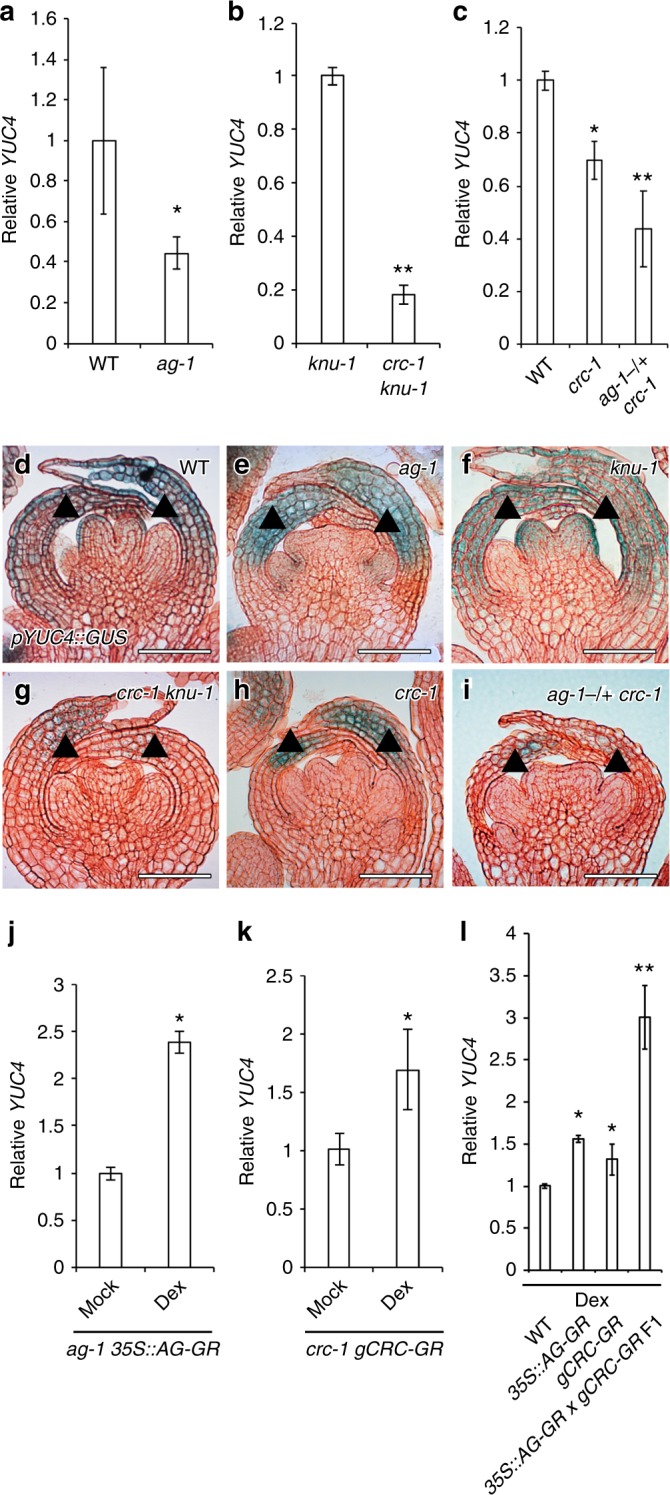
AG and CRC synergistically control *YUC4* expression. **a**–**c** *YUC4* mRNA levels in wild-type and *ag-1* (**a**), *knu-1* and *crc-1 knu-1* (**b**), wild-type, *crc-1*, and *ag-1-/* *+* *crc-1* (**c**) floral buds. The values are represented as the means ± SEMs. *p*-values were calculated with a two-tailed Student’s *t*-test. *p < 0.05 compared to wild type. **p < 0.05 compared to single mutant. **d**–**i** *pYUC4::GUS* in wild-type (**d**), *ag-1* (**e**), *knu-1* (**f**), *crc-1 knu-1* (**g**), *crc-1* (**h**), and *ag-1-/* *+* *crc-1* (**i**) floral buds at the stage 6. Arrowheads indicate abaxial side of epidermal cells in carpels at stage 6. **j**–**l** *YUC4* mRNA levels in mock- and dex-treated *ag-1 35* *S::AG-GR* (**j**), mock- and dex-treated *crc-1 gCRC-GR* (**k**), dex-treated wild-type, *35S::AG-GR*, *gCRC-GR*, and *35S::AG-GR gCRC-GR* F1 (**l**). The values are represented as the means ± SEMs. *p*-values were calculated with a two-tailed Student’s *t*-test. *p < 0.05 compared to mock or wild type. **p < 0.05 compared to single transgenic line. Bar = 50 µm in **d**–**i**

We performed a quantitative reverse transcription-polymerase chain reaction (qRT-PCR) analysis to confirm the microarray results using total RNA extracted from floral bud clusters up to stage 7. Similar to previous microarray data, we also detected a reduction in *YUC4* transcript levels in the *ag-1* mutant compared with the wild type (Fig. 2a). *YUC4* mRNA levels were also lower in *crc knu* than in *knu* (Fig. 2b). Low levels of *YUC4* expression were also found in the *crc* single mutant (Fig. 2c). A further reduction in *YUC4* transcript levels was observed in the *ag-1-/+ crc-1* mutant (Fig. 2c), which was accompanied by a reduction in floral meristem determinacy, when compared to the *crc* single mutant as previously reported^12^ (Supplementary Fig. 3). In addition, the expression of the stem cell determinant, *WUS*, increased in the *ag-1-/+ crc-1* mutant relative to the *crc-1* mutant (Supplementary Fig. 3).

To investigate *YUC4* expression, we generated a *pYUC4::GUS* line. The 3.7 kb upstream of the *YUC4* promoter recapitulated the *YUC4* mRNA distribution pattern: highest signals were detected in the abaxial side of epidermal cells in carpels at stage 6 in both the independent T1 *YUC4* reporter lines and by *YUC4* mRNA in situ hybridization (Supplementary Fig. 4). At stage 7, *YUC4* expression was restricted to the tip of carpels (Supplementary Fig. 4). This expression pattern is similar to that reported previously^47^. A spatial expression test revealed that the expression domains of the *CRC* mRNA, CRC protein, and *YUC4* reporter overlapped in the abaxial carpels at stage 6 of flower development in the wild type (Fig. 2d and Supplementary Fig. 5). Although we detected the effects of *ag* or *crc* mutations on *YUC4* expression using floral bud clusters up to stage 7 by RT-PCR, the observed expression patterns could be due to secondary effects such as tissue composition differences. We used two different approaches to confirm the contribution of AG and CRC to *YUC4* transcription. In the first approach, we examined the spatial expression patterns of *YUC4* in the mutant background at stage 6, where tissue composition differences are minimal. In the *ag-1* mutant, *YUC4* was mostly limited to the presumptive nectaries at the base of the third whorl (Fig. 2e). While the *YUC4* signal intensity and expression pattern was similar between *knu-1* and the wild type, *YUC4* expression in the *crc-1 knu-1* and *crc-1* mutants was reduced specifically in the abaxial carpels at stage 6 of flower development in the wild type (Fig. 2f–h). Reduction of auxin synthesis genes in *crc-1* could be dependent on *YUC4* since *TAA1* expression was not affected in *crc-1* as in the wild type^24^ (Supplementary Fig. 6). Furthermore, *YUC4* expression was lower in *ag-1*-/+ *crc-1* than in the wild type (Fig. 2i). Although *YUC4* is expressed in sepals, no difference in *YUC4* expression was observed in any of the mutants tested here, suggesting that *YUC4* expression in sepals is not regulated by AG or CRC.

In the second approach used to examine the roles of AG and CRC in regulating *YUC4* expression, we monitored *YUC4* expression after a short induction of AG and/or CRC. We expressed AG or CRC proteins fused to a glucocorticoid receptor hormone binding domain (GR). Nuclear entry of the AG-GR fusion protein by dexamethasone (dex) treatment in an *ag* mutant background^48^ triggered a 2.5-fold increase in *YUC4* expression within 4 h (Fig. 2j). Likewise, CRC-GR induction by dex treatment in a *crc* mutant background^17^ triggered *YUC4* expression (Fig. 2k). Furthermore, simultaneous activation of AG and CRC by dex in AG-GR CRC-GR double transgenic plants led to a significant increase in *YUC4* expression compared to either of the parental lines (Fig. 2l). Taken together, these data suggest that AG and CRC synergistically regulated *YUC4* expression.

### CRC binds to *YUC4* promoter and promotes auxin accumulation

**Fig. 3:**
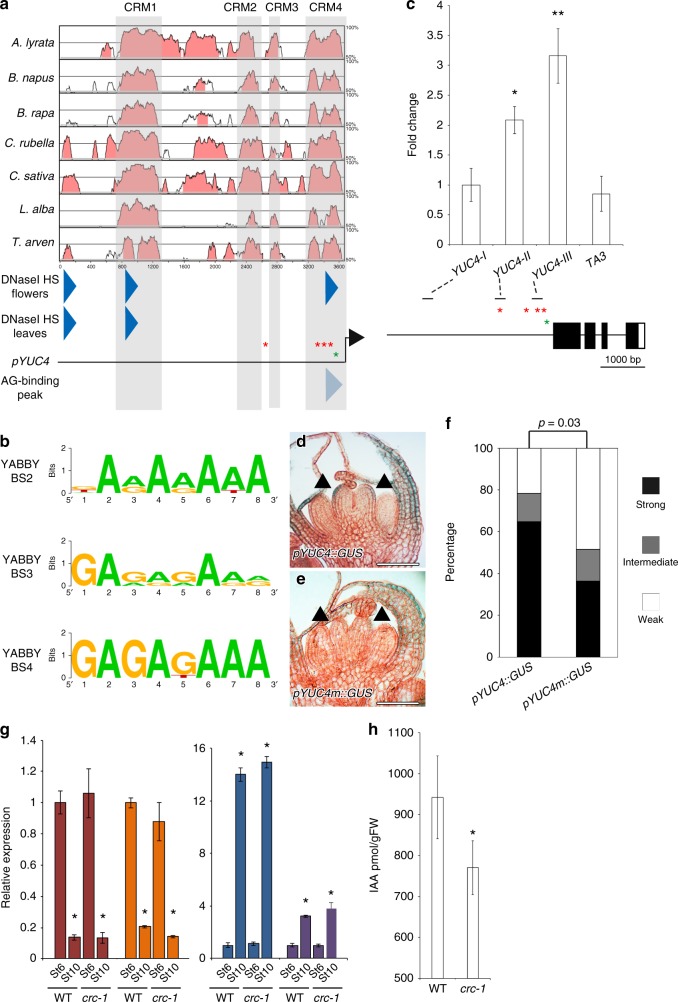
CRC binds to the *YUC4* promoter and controls auxin accumulation **a** Pairwise alignment created by mVISTA of the 5′ upstream intergenic region of the *YUC4* promoter using seven *Brassicaceae* species. Four regions (gray: CRM1, CRM2, CRM3, and CRM4) are conserved in the *YUC4* promoter. Blue triangles indicate DNase I hypersensitive sites. Red and green asterisks indicate CRC binding sites and a possible CArG box (C[C/T][A/T]G[A/G][A/T]_6_[A/G]G) within an AG binding peak, respectively. The binding region was shown by pale blue triangle. **b** Conservation of CRC binding sites among *Brassicaceae* species. The diagram was generated by Weblogo. **c** Mapping of the CRC-myc protein to the *YUC4* locus. ChIP using anti-myc in synchronized *gCRC-myc* floral buds at stage 6 in the *ap1 cal* background. The bottom section indicates the location of ChIP-qPCR amplicons tested. Red and green asterisks indicate CRC binding sites and possible CArG box, respectively. The values are represented as the means ± SEMs. *p*-values were calculated with a two-tailed Student’s *t*-test. *p < 0.05 compared to the *TA3* signal. **p < 0.01 compared to the *TA3* signal. **d**, **e** Reporter gene expression in the wild-type and YABBY-binding site-mutated *YUC4* promoter. *GUS* expression in stage 6 floral buds under control of the *YUC4* promoter with (**d**) or without (**e**) intact YABBY-binding sites. Arrowheads indicate abaxial side of epidermal cells in carpels at stage 6. **f** Visual scoring of *pYUC4::GUS* or *pYUC4m::GUS* staining in the T1 population; *n* > 32 for each construct tested. *p*-values were calculated with a χ^2^ *t*-test. **g** mRNA abundance of marker genes, *CLV3* (red), *LFY* (orange), *PAL4* (blue), and *COMT* (purple) in wild-type and *crc-1* floral buds up to stage 6 and stage 10. The values are represented as the means ± SEMs. *p*-values were calculated with a two-tailed Student’s *t*-test. *p < 0.05 compared to the signals at stage 6 in the same genetic backgrounds. **h** The amount of IAA in WT and *crc-1* mutants determined by liquid chromatography-tandem mass spectrometry. Floral buds up to stage 8 were used for this assay. The values are represented as the means ± SEMs. *p*-values were calculated with a two-tailed Student’s *t*-test. *p < 0.05 compared to the wild type. Bars = 50 µm in **d**, **e**

Our combined results suggest that CRC may directly upregulate *YUC4*. To identify candidate YABBY-binding sites in the *YUC4* promoter, we first performed phylogenetic shadowing with seven *Brassicaceae* species^15,17^. Four conserved regulatory modules (CRMs) were identified and defined as CRM1, CRM2, CRM3, and CRM4 (Fig. 3a and Supplementary Fig. 7–9). Then, we identified DNase I hypersensitive sites where transcription factors could bind using published genome-wide data^49^ (Fig. 3a and Supplementary Fig. 10). CRM1 contained a DNase I hypersensitive site in the leaves and flowers, suggesting that CRM1 had a key role in regulating *YUC4* expression in both vegetative and reproductive stages (Fig. 3a). CRM4 also contained a DNase I hypersensitive site that was observed only in the reproductive stage (Fig. 3a). Furthermore, an AG binding peak was in the CRM4. Within the peak, there is only one potential CArG box, where AG could bind (Fig. 3a). Since CRC is specifically expressed during the reproductive stage^17^, CRM4 might have an important role in activating *YUC4* expression. Consistent with this hypothesis, the deletion of CRM4 by genome editing led to partial reduction in *YUC4* mRNA levels compared with the parental line (Supplementary Fig. 9). Remaining activity of *YUC4* mRNA in the deletion line could be due to the presence of regulatory region other than CRM4. CRM4 contained three evolutionarily conserved YABBY-binding sites (GA[A/G]AGAAA) (Fig. 3a, b, and Supplementary Fig. 9). Two of these sites were highly conserved among all seven *Brassicaceae* species while the other was partially conserved only in four species (BS2). Although a fourth YABBY-binding site was observed in CRM2 and CRM3, it was not conserved. No other potential YABBY-binding sites (CC[C/A][T/C]C[T/A][C/T]C or CCCCAC) were found anywhere in the *YUC4* promoters among the seven *Brassicaceae* species^50^ (Supplementary Fig. 7-10). Furthermore, no flower-specific DNase I hypersensitive site containing YABBY-binding sites was found in the regulatory region of the three closest *YUC4* homologs^25^ (*YUC1*, *YUC2*, and *YUC6*) (Supplementary Fig. 10).

To determine whether CRC directly binds to the *YUC4* promoter, we used a genomic construct that expressed a CRC-myc fusion protein (gCRC-myc). Because of limited floral tissues at a specific stage, a ubiquitously expressed APETALA1 (AP1) fusion to GR in the *ap1 cal* mutant background was employed. The *ap1 cal* double mutant formed many floral primordia at the first and second developmental stages. The dex-induced nuclear entry of AP1-GR in the *ap1 cal* mutant resulted in the production of hundreds of floral stage-synchronized tissues^51^. Chromatin immunoprecipitation (ChIP) using synchronized CRC-myc flowers at stage 6 followed by qPCR revealed a strongest association of CRC-myc with the *YUC4-III* DNA fragment, which contained three evolutionarily conserved YABBY-binding sites found in CRM4 (Fig. 3a, c).

To test the biological functions of the YABBY-binding sites in the CRM4, all three YABBY-binding sites were disrupted in a full-length *YUC4* promoter (*pYUC4*), yielding *pYUC4m*. In transgenic plants expressing *GUS* under control of *pYUC4m, GUS* expression was specifically reduced in abaxial carpels at stage 6 of flower development (Fig. 3d, e). We analyzed the expression of these reporter constructs in an independent population of T1 plants (*n* ≥ 32) to minimize potential positional effects on transgene expression. Significantly more *pYUC4m::GUS* lines had weaker signals than *pYUC4::GUS* lines (*p* *=* 0.03) (Fig. 3f). The combined data suggest that CRC directly binds to the *YUC4* promoter via highly conserved *cis*-elements.

Then, we quantified bioactive auxin to examine the contribution of the CRC-induced increase in *YUC4* transcription to auxin accumulation. Since *CRC* expression began in stage 6 and no morphological differences were observed between the wild type and *crc* single mutant at this stage^13^, flowers older than the stage 7 were manually selected for and removed using a dissecting microscope (Supplementary Fig. 11a-d). To evaluate the quality of these tissues, qRT-PCR was conducted. The expression of a stem cell marker gene (*CLAVATA3; CLV3*) and a young flower-specific gene^52,53^ (*LEAFY; LFY*) were higher in stage 6 floral buds than in stage 10 buds (Fig. 3g, and Supplementary Fig. 11e). By contrast, the transcript levels of genes involved in monolignol and cellulose biosynthesis, such as *PHENTLALANINE AMMONIA-LYASE4* (*PAL4*) and *CAFFEATE O-METHYLTRANSFERASE1* (*COMT1*), were very low in stage 6 tissues compared to stage 10 tissues^54^ (Fig. 3g and Supplementary Fig. 11e). We used well-trimmed tissues to measure the amount of indole-3-acetic acid (IAA). Consistent with the observed reduction in *YUC4* transcripts in the *crc-1* mutant, our analysis of IAA levels by mass spectrometry after liquid chromatography revealed that IAA levels were reduced in the *crc* mutants (Fig. 3h).

### The AG complex controls chromatin accessibility at *YUC4*

**Fig. 4:**
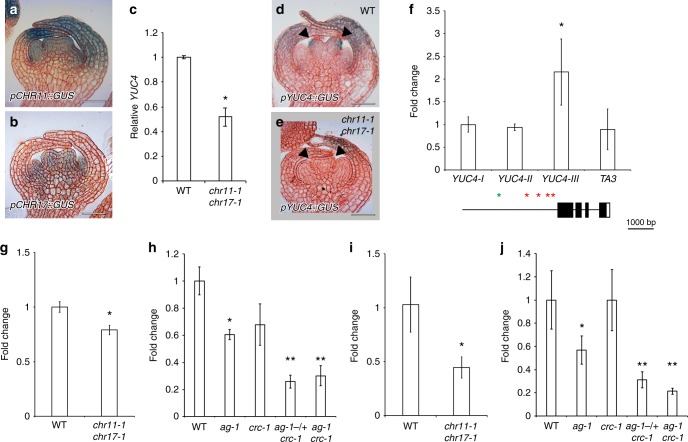
AG and CRC are required for increased chromatin accessibility at the *YUC4* loci. **a**, **b** Expression of *pCHR11*::*GUS* (**a**) and *pCHR17*::*GUS* (**b**) in stage 6 floral buds. **c** *YUC4* mRNA levels in wild-type and *chr11-1 chr17-1* floral buds. The values are represented as the means ± SEMs. *p*-values were calculated with a two-tailed Student’s *t*-test. *p < 0.05 compared to the wild type. **d**, **e** Expression of *pYUC4::GUS* in wild-type (**d**) and *chr11-1 chr17-1* (**e**) floral buds at the stage 6. Arrowheads indicate abaxial side of epidermal cells in carpels at stage 6. **f** Top: Mapping of the GFP-CHR11 protein association to the *YUC4* locus. A GFP antibody was used to detect CHR occupancy. Bottom: Diagram of ChIP-qPCR amplicons tested. Red and green asterisks indicate CRC binding sites and possible CArG box (C[C/T][A/T]G[A/G][A/T]6[A/G]G) within an AG binding peak, respectively. The values are represented as the means ± SEMs. *p*-values were calculated with a Student’s *t*-test. *: *p* < 0.05 compared to the *TA3* signal. **g**, **h** Chromatin accessibility at the *YUC4* locus. **g**) Wild-type and *chr11-1 chr17-1* floral buds. **h**) Wild-type, *ag-1*, *crc-1*, *ag-1-/* *+* *crc-1*, and *ag-1 crc-1* floral buds. The values are represented as the means ± SEMs. *p*-values were calculated with a two-tailed Student’s *t*-test. *p < 0.05 compared to the wild type. **p < 0.05 compared to single mutants. **i**, **j** RNA Pol II association with the *YUC4* locus. A Pol II antibody was used. **i** Wild-type and *chr11-1 chr17-1* floral buds. **j** Wild-type, *ag-1*, *crc-1*, *ag-1-/+ crc-1*, and *ag-1 crc-1* floral buds. The values are represented as the means ± SEMs. *p*-values were calculated with a two-tailed Student’s *t*-test. *p < 0.05 compared to the wild type. **p < 0.05 compared to single mutants. Bars = 50 µm in **a**, **b**, **d**, **e**

One possible explanation for the synergistic activation of *YUC4* by AG and CRC could be a physical protein–protein interaction between AG and CRC. However, no interaction was observed between the two transcription factors based on yeast-two hybrid (Supplementary Fig. 12). Alternatively, chromatin accessibility at the *YUC4* promoter may be affected. Since the imitation switch (ISWI)-type chromatin remodeling factors CHROMATIN REMODELING 11 (CHR11) and CHR17 were identified as AG interacting partners and a *chr11 chr17* double mutant showed a split style phenotype^55–58^ (Supplementary Fig. 13), we examined the role of AG and CHR11/CHR17 in regulating *YUC4*. We treated *chr11 chr17* double mutants with exogenous auxin; however, no rescue was observed under our experimental conditions (Supplementary Fig. 13). Local accumulation of auxin is important for phenotypic rescue in *chr11 chr17* mutants as is often seen in many mutants^25^. *CHR11* and *CHR17* were both expressed in terminating floral meristems at stage 6 (Fig. 4a, b). Based on qRT-PCR analysis, *YUC4* expression was reduced in the *chr11-1 chr17-1* double mutant compared to the wild type (Fig. 4c). We confirmed the reduction in *YUC4* expression in the carpels of the *chr11-1 chr17-1* double mutant at stage 6 (Fig. 4d, e). CHR11 bound to the proximal region of the *YUC4* promoter, similar to AG (Fig. 4f and Supplementary Fig. 14).

The primary function of CHR11 and CHR17 is to slide nucleosomes and open up chromatin to allow proper gene expression^57,59^. Once genes are transcriptionally activated, nucleosome occupancy typically decreases^60^. Published genome-wide nucleosome distribution in the wild type revealed that there was one nucleosome upstream (−1 nucleosome) and several well-positioned nucleosomes downstream of the *YUC4* transcriptional start site (TSS) (Supplementary Fig. 15)^57^. Consistent with the observed reduction in *YUC4* expression in the *chr11 chr17* double mutant, published MNase-seq data^57^ show a higher nucleosome occupancy (+1 nucleosome) on the TSS of *YUC4* compared to the wild type (Supplementary Fig. 15). To further examine the effect of CHR11/CHR17, AG, and CRC on the accessibility of the *YUC4* locus, we performed Formaldehyde Assisted Isolation of Regulatory Elements (FAIRE)^61^. The FAIRE assay revealed a reduction in open chromatin at the *YUC4* promoter in the *chr11 chr17* double mutant compared to the wild type (Fig. 4g). Likewise, the *ag* mutation decreased accessibility to *YUC4* based on FAIRE (Fig. 4h). On the other hand, we did not see statistic difference in chromatin accessibility between wild type and *crc* mutant (Fig. 4h). A further reduction in chromatin accessibility was observed in the *ag-1-/+ crc-1* heterozygote and *ag-1 crc-1* double mutant compared to either single mutant (Fig. 4h). RNA polymerase II (Pol II) occupancy was also reduced in the *chr11 chr17* mutant relative to the wild type (Fig. 4i). A similar reduction in Pol II occupancy was observed in the *ag-1* mutant relative to the wild type (Fig. 4j). By contrast, no difference in Pol II occupancy was observed in the *crc-1* mutant (Fig. 4j), and a subtle reduction was observed in the *ag-1* mutant compared with the wild type. In addition, a further reduction was observed in the *ag-1-/+ crc-1* heterozygote and the *ag-1 crc-1* double mutant compared to the parental lines (Fig. 4j). Taken together with expression analyses, our results may suggest that the AG-CHR11/CHR17 complex controls chromatin-mediated accessibility to the *YUC4* promoter and thereafter CRC affects the chromatin structure through transcriptional regulation.

### *YUC4* misexpression rescues the indeterminacy in *crc knu*

**Fig. 5:**
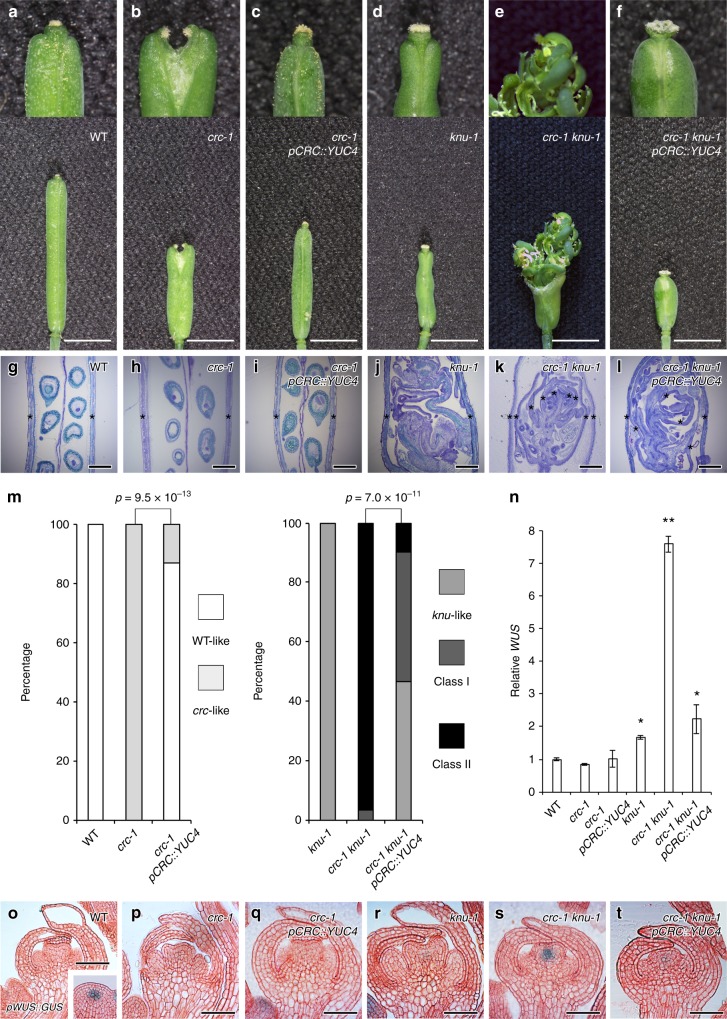
Rescue of indeterminacy in *crc knu* by ectopic expression of *YUC4*. **a**–**f** Morphology of wild-type (**a**), *crc-1* (**b**), *crc-1 pCRC::YUC4* (**c**), *knu-1* (**d**), *crc-1 knu-1* (**e**), and *crc-1 knu-1 pCRC::YUC4* (**f**) fruits. Above: Close-up views of fruit tips. Below: Shapes of whole fruits. Arrowheads indicate stigma structures. **g**–**l** Longitudinal section of wild-type (**g**), *crc-1* (**h**), *crc-1 pCRC::YUC4* (**i**), *knu-1* (**j**), *crc-1 knu-1* (**k**), and *crc-1 knu-1 pCRC::YUC4* (**l**) fruits. Asterisks indicate carpels. **m** Quantification of the mutant phenotype. *p*-values were calculated with a Chi-Square test. **n** Expression of the stem cell marker *WUS* in wild-type, *crc-1, crc-1 pCRC::YUC4, knu-1*, *crc-1 knu-1*, and *crc-1 knu-1 pCRC::YUC4* flowers. The values are represented as the means ± SEMs. *p*-values were calculated with a two-tailed Student’s *t*-test. *p < 0.05 compared to the wild type. **p < 0.05 compared to the *knu-1* mutants. **o**–**t** Expression of the stem cell marker *WUS* in longitudinal sections of wild-type (**o**), *crc-1* (**p**), *crc-1 pCRC::YUC4* (**q**), *knu-1* (**r**), *crc-1 knu-1* (**s**), and *crc-1 knu-1 pCRC::YUC4* (**t**) stage 6 floral buds. Images are shown at the same magnification. Bars = 1 cm in **a**–**f**; 500 μm in **g**–**l**; 50 μm in **o**–**t**

Since *YUC4* expression is directly controlled by CRC, we then investigated whether expressing *YUC4* was sufficient to rescue the *crc* mutant phenotype (Fig. 5a–n). We expressed *YUC4* under the control of the *CRC* promoter in the *crc* mutant and sensitized *crc knu* mutant. As previously reported^13,17^. *crc* mutants had shorter and wider fruits than the wild type (Fig. 5a, b, g, h). Furthermore, *crc* mutants failed to fuse at the fruit tips and had shorter styles than the wild type (Fig. 5a, b, g, h, m). The *crc* mutant phenotype was significantly rescued by introducing *YUC4* under the control of the *CRC* promoter (*p* = 9.5 × 10^−13^; Fig. 5b, c, h, i, m). The *knu-1* and *crc-1 knu-1* mutants formed bulged gynoecia that contained additional whorls of carpels, and the indeterminate shoot phenotype, respectively^17^ (Fig. 5d, e, j, k). When *crc knu* was crossed with *pCRC::YUC4*, the resulting plants (*pCRC::YUC4 crc knu*) did not exhibited outgrowth of the indeterminate shoot phenotype (Fig. 5d–f, j–l). Misexpression of *YUC4* under control of the *CRC* promoter in the *crc knu* mutant significantly rescued the effect of the *crc* mutation (*p* = 7.0 × 10^−11^): approximately half of the *pCRC::YUC4 crc-1 knu-1* fruits showed a *knu* mutant-like phenotype. These results indicated that local overproduction of *YUC4* in the *CRC* expression domain was sufficient to restore the *crc knu* mutant back to the *knu* phenotype.

To investigate the effect of *YUC4* misexpression on the floral meristem indeterminacy phenotype at molecular level, we examined the expression of a stem cell determinant gene, *WUS*^7^ (Fig. 5n–t). Although a strong *pWUS::GUS* signal was detected in the inflorescence meristem in wild-type plants, we could not detect the signal in the wild type or *crc-1* floral buds at stage 6 (Fig. 5n–p). No *WUS* signal was observed in *crc-1 pCRC::YUC4*, which was consistent with the phenotypic similarity with wild-type plants (Fig. 5n, o, q). In *knu* and *crc knu* stage 6 mutant flowers, *WUS* was either weakly or strongly misexpressed, respectively^11,17^ (Fig. 5n, r, s). The strong *WUS* expression in *crc knu* was compromised when we introduced *pCRC::YUC4* (Fig. 5n, s, t). Our combined data implied that *YUC4* misexpression restored the *crc knu* mutant phenotype and *WUS* expression to that of the *knu* mutant.

### *YUC4* misexpression rescues the cell wall defects in *crc knu*

**Fig. 6:**
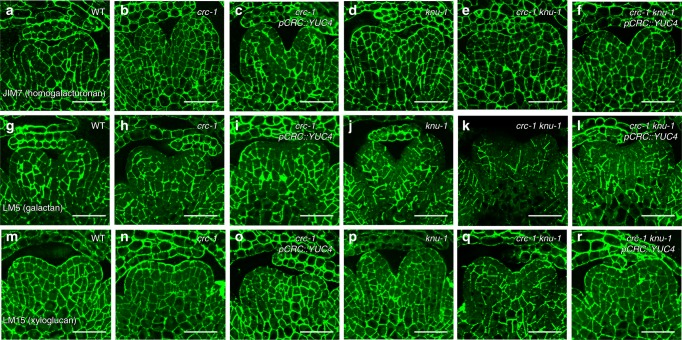
Rescue of galactan and xyloglucan distribution in the *crc knu* by ectopic expression of *YUC4*. **a**–**f** Immunolocalization of homogalacturonan epitopes with JIM7 using longitudinal sections of wild-type (**a**), *crc-1* (**b**), *crc-1 pCRC::YUC4* (**c**), *knu-1* (**d**), *crc-1 knu-1* (**e**), and *crc-1 knu-1 pCRC::YUC4* (**f**) stage 6 floral buds. **g**–**l** Immunolocalization of (1-4)-b-d-galactan epitopes with LM5 using longitudinal sections of wild-type (**g**), *crc-1* (**h**), *crc-1 pCRC::YUC4* (**i**), *knu-1* (**j**), *crc-1 knu-1* (**k**), and *crc-1 knu-1 pCRC::YUC4* (**l**) stage 6 floral buds. **m**–**r** Immunolocalization of xyloglucan epitopes with LM15 using longitudinal sections of wild-type (**m**), *crc-1* (**n**), *crc-1 pCRC::YUC4* (**o**), *knu-1* (**p**), *crc-1 knu-1* (**q)**, and *crc-1 knu-1 pCRC::YUC4* (**r**) stage 6 floral buds. Bars = 50 µm in **a**–**r**

A microarray analysis suggested that floral meristem termination could be associated with cell wall remodeling (Fig. 1). To examine the effect of the AG-CRC-YUC4 pathway on cell wall components during floral meristem termination, we probed stage 6 floral bud sections with antibodies that recognized specific plant cell wall components. In the wild-type inflorescence meristem and floral meristem, immunolocalization of methyl esterified homogalacturonan (HG) with JIM7 exhibited a uniform labeling pattern over the different floral organs throughout cell walls^30^ (Fig. 6a and Supplementary Fig. 16). No difference was found in methyl esterified HG distribution using JIM7 between the mutants or transgenic plants and the wild type (Fig. 6a–f).

LM5 specifically recognizes (1-4)-β-d-galactan. As previously reported on the inflorescence meristems, this antibody binds weakly to the L1 layer cells of terminating floral meristems in the wild type^30^ (Fig. 6g and Supplementary Fig. 16). A similar LM5 labeling pattern and signal intensity was observed in *crc-1*, *crc-1 pCRC::YUC4*, and *knu-1* (Fig. 6h–j), and the LM5 labeling intensity was reduced in the *crc-1 knu-1* double mutants (Fig. 6k). Consistent with the phenotypic rescue of *crc knu* by *YUC4* misexpression, labeling intensity with LM5 in *crc knu pCRC::YUC4* was also restored (Fig. 6l, Supplementary Fig. 16). Xyloglucan recognized by LM15 was weakly observed in the L1 layer of the wild-type inflorescence meristem, where it accumulated uniformly in the developing carpels^30^ (Fig. 6m and Supplementary Fig. 16). A similar LM15 binding pattern was observed in *crc-1*, *knu-1*, and *pCRC::YUC4* (Fig. 6n–p); however, xyloglucan accumulation in the L1 layer of the developing *crc-1 knu-1* carpels was weaker than in the wild type (Fig. 6q). This reduction was also partially restored by ectopic expression of *YUC4* (Fig. 6r). Since we did not observe differences in the thickness of the cell wall in any carpel primordia among the tested lines, alterations in our antibody labeling experiment were largely due to changes in cell wall composition (Supplementary Fig. 17).

### *YUC4* and *TRN2* regulate floral meristem determinacy

**Fig. 7:**
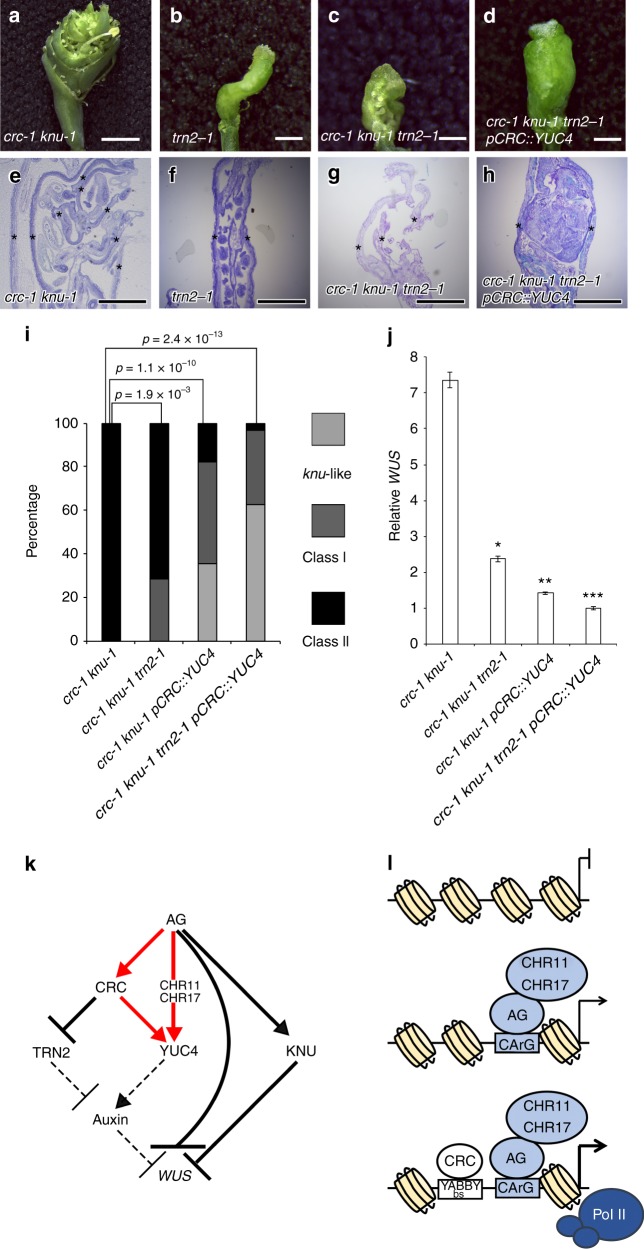
Rescue of the *crc knu* mutant phenotype by modulating two CRC downstream target genes. **a**–**d** Morphology of *crc-1 knu-1* (**a**), *trn2-1* (**b**), *trn2-1 crc-1 knu-1* (**c**), and *trn2-1 crc-1 knu-1 pCRC::YUC4* (**d**) fruits. **e**–**h** Longitudinal section of *crc-1 knu-1* (**e**), *trn2-1* (**f**), *trn2-1 crc-1 knu-1* (**g**), and *trn2-1 crc-1 knu-1 pCRC::YUC4* (**h**) fruits. Asterisks indicate carpels. **i** Quantification of mutant phenotypes of *crc-1 knu-1*, *crc-1 knu-1 trn2-1*, *crc-1 knu-1 pCRC::YUC4* and *crc-1 knu-1 trn2-1 pCRC::YUC4* fruits. *p*-values were calculated with a Chi-Square test. **j** Expression of the stem cell marker *WUS* in *crc-1 knu-1*, *crc-1 knu-1 trn2-1*, *crc-1 knu-1 pCRC::YUC4*, and *crc-1 knu-1 trn2-1 pCRC::YUC4* flowers. *p*-values were calculated with a two-tailed Student’s *t*-test. *p < 0.05 compared to *crc knu*. **p < 0.05 compared to *crc knu trn2*. ***p < 0.05 compared to *crc knu pCRC::YUC4*. **k** Regulatory network for AG-mediated floral meristem termination in *Arabidopsis*. Red arrows: feed-forward regulation of the *YUC4* by AG and CRC. Solid arrows: direct transcriptional regulation. Dashed arrows: relationship may not be direct. **l** A model for the *YUC4* transcription. Bars = 3 mm in **a**; 1 mm in **b**–**d**; 500 μm in **e**–**h**

To examine whether *YUC4* and *TRN2* both control floral meristem determinacy downstream of CRC, we ectopically expressed *YUC4* and removed TRN2 activity in the *crc knu* double mutant background (Fig. 7a–l). Although *trn2* fruits were twisted, the mutant did not show defects in floral meristem determinacy (Fig. 7b, f). The effect of the *crc* mutation on floral meristem indeterminacy in the *knu* mutant background was partially restored by the introduction of the *trn2* mutation (*p* = 1.9 × 10^−3^) (Fig. 7a–c, e–g, i) as previously reported^17^. Activation of *YUC4* in the *crc knu* mutant background also partially restored the mutant phenotype to that of the *knu* mutant (*p* = 1.1 × 10^−10^). Simultaneous activation of *YUC4* and removal of *TRN2* led to a dramatic rescue of the effect of the *crc* mutation to that of the *knu* mutant background (*p* = 2.4 × 10^−13^) (Fig. 6d, h, i). Less than 5% of the *crc knu trn2 pCRC::YUC4* plants showed the strong *crc knu* mutant phenotype. Most *crc-1 knu-1 trn2-1 pCRC::YUC4* fruits showed the *knu-1* phenotype in terms of floral meristem determinacy (Fig. 7d, h, i). The *crc-1 knu-1 trn2-1 pCRC::YUC4* fruits never showed unclosed fruits phenotype which often seen in *crc-1 knu-1 trn2-1*. Inside structure in *crc-1 knu-1 trn2-1 pCRC::YUC4* and *knu-1* was similar to each other (Figs. 5j and 7h).

To test whether the phenotypic changes seen in *crc-1 knu-1 trn2-1 pCRC::YUC4* were associated with *WUS* expression, we examined *WUS* expression by qRT-PCR. The strong *WUS* expression in the *crc knu* double mutants was attenuated by either activation of *YUC4* or removal of *TRN2*^17^ (Fig. 7j). We observed a further reduction of *WUS* expression in *crc-1 knu-1 trn2-1 pCRC::YUC4* compared to *crc-1 knu-1 trn2-1* or *crc-1 knu-1 pCRC::YUC4*. Hence, the two direct CRC targets, *YUC4* and *TRN2*, cooperatively contribute to the termination of floral meristem development through *WUS* regulation (Fig. 7k).

## Discussion

The timing of floral meristem termination is critical to forming a female reproductive structure (gynoecium). Despite the importance of gynoecium development for reproductive success, little is known about the switch from floral stem cell maintenance to gynoecium formation that is induced by the plant hormone auxin. Here, we showed that the auxin biosynthesis gene, *YUC4*, contributes to the transition from floral stem cell maintenance to gynoecium formation in *Arabidopsis*. Two tissue-specific transcription factors, AG and CRC, bind directly to the *YUC4* promoter and activate its expression. Positive transcriptional feed-forward loops, where a transcription factor directly activates another transcription factor, have been observed during flower development and auxin synthesis^62,63^. Although the CArG box and YABBY-binding sites (CRC binding site) are located close to each other on the *YUC4* promoter, we did not observe protein–protein interactions between AG and CRC. Instead, we found that feed-forward activation of *YUC4* expression by AG and CRC directs the coordination of a precise change in chromatin state. This feed-forward loop may be important to overcome polycomb-mediated silencing and to reactivate the *YUC4* gene only in correct timing and place^64^. Previous proteomics approaches identified some nucleosome-associated factors that physically interact with AG^58^. Among these nucleosome-associated factors, SWI2/SNF2 chromatin remodeling ATPase is known to alter the interaction between the histone octamer and the DNA using ATP hydrolysis for energy^65^. The ISWI-type chromatin remodeling factors CHR11 and CHR17 were shown to fuse carpels^58^. Since *ag* and *chr11 chr17* mutants displayed similar levels of *YUC4* expression, Pol II binding, and chromatin structure, CHR11 and CHR17 may enable AG to activate its targets. Since previous studies and our analyses agree that expression of *AG* and *CHR11/CHR17* overlap during flower development stage 3^3,56^ (Supplementary Fig. 18), we propose that the AG-CHR chromatin remodeling complex opens up compacted chromatin, thereby increasing accessibility to the *YUC4* gene prior to floral meristem termination. Although *YUC4* expression was reduced in the *ag* mutant, no difference was reported in floral meristem size between the wild type and *ag* mutant by stage 3^66^. This could be due to redundant activity within the *YUC* gene family or other auxin-related regulators such as TRN2 (see below). Except for *YUC4*, none of the other *YUC* family members have been identified as direct AG targets according to previous genome-wide AG binding data^40^. We also did not detect any differences in the expression of other *YUC* genes between the wild type and *ag-1* mutants (Supplementary Fig. 19).

Because *CRC* expression starts in stage 6 of flower development, it is most likely that CRC binds to the *YUC4* promoter after the AG-CHR11/CHR17 complex opens up the *YUC4* locus^3,13,57,58^. Thus, a synergistic activation of *YUC4* is more likely to occur where and when both *AG* and *CRC* are expressed. High expression of *YUC4* was only detected in the abaxial region of carpel primordia at stage 6. At the molecular level, the AG-CHR chromatin remodeling complex may allow additional transcription factors, such as CRC, to access their respective *cis*-elements. This could lead to further recruitment of general transcriptional machinery, including RNA Pol II and possibly other Pol II complex components, to boost transcription of *YUC4* (Fig. 7l). Although we did not observe a difference in *AG* expression between the wild type and *chr11-1 chr17-1* double mutant, *CRC* expression was decreased in the *chr11-1 chr17-1* double mutant (Supplementary Fig. 13). Thus, the AG-CHR11/CHR17 complex might control *CRC* expression in addition to *YUC4* expression. The synergistic feed-forward regulation of *YUC4* expression by AG and CRC directs the precise chromatin state switch from floral stem cell maintenance to gynoecium formation through auxin. Furthermore, auxin maxima are likely established in the terminating floral meristem during stage 6, together with other auxin synthesis enzymes (YUCs, TAA) and the CRC-mediated auxin homeostasis regulator TRN2^17,24^. Indeed, the indeterminate phenotype of the *yuc4* mutant was only observed in the sensitized *yuc* multiple mutant background^25^.

Previously, we reported that CRC terminates floral meristem through the auxin homeostasis pathway^17^. The CRC downstream target, *TRN2*, contributes to this termination, at least partially^17^. Here, we showed that two auxin-related CRC targets, *YUC4* and *TRN2*, function in a parallel pathway for the termination of floral meristem. In the *crc knu* double mutant, *YUC4* expression decreased, whereas *TRN2* expression increased. Ectopic expression of *YUC4* and removal of *TRN2* in the *crc knu* background resulted in a phenotype similar to that of the *knu* mutant. Since we observed significant rescue of floral meristem determinacy (that had been disrupted by the *crc* mutation) in the sensitized *knu* mutant background, these two genes could be the major auxin homeostasis targets that act downstream of CRC.

We also noticed a difference in the accumulation of galactan and xyloglucan in the cell wall of the *crc knu* double mutant compared to the wild type. Galactan accumulated at lower levels in the L1 layer in the wild-type shoot apical meristem and terminating floral meristem at the stage 6. This suggested that the high mobility of galactan in the cell wall and/or reversible binding of galactan to cellulose in a layer-specific manner might be important for proper floral meristem function, as was previously suggested for the shoot apical meristem^67^. In addition to the defects in floral meristem activity, galactan levels were also reduced in all three floral meristem layers of the *crc knu* double mutant. Xyloglucan also accumulated in a layer-specific manner in the wild-type shoot apical meristem, but not in the wild-type floral meristem at stage 6^30^. Interestingly, tissue-specific accumulation of xyloglucan was only observed in the *crc knu* floral meristem at the stage 6. This finding suggests that cellular morphogenetic events might be similar between the wild-type shoot apical meristem and the *crc knu* mutant floral meristem. Our antibody labeling of wall components suggests a potential role for galactan and xyloglucan rearrangements in floral meristem termination. Furthermore, alterations in cell wall composition in the *crc knu* mutant were partially rescued when *YUC4* was misexpressed. These data suggest that the cell wall composition downstream of CRC might be regulated by auxin. A second possibility is that this rescue reflects the ability of auxin biosynthesis to partially bypass defects in cell wall composition. In this scenario, CRC target candidates, such as *EXP8* and *AtcwINV1*, could have important roles in cell wall composition.

## Methods

### Plant materials and growth condition

All plants used in this study were in the Landsberg *erecta* (L*er*) background, except for *chr11-1 chr17-1* and *pTAA1::TAA1-GFP*, which were in the Columbia (Col) background. The *ag-1*, *crc-1*, *knu-1*, *crc-1 knu-1*, *crc-1 gCRC-GR*, *ag-1 35S::AG-GR*, *ap1 cal-1 35S::AP1-GR gCRC-myc*, *chr11-1 chr17-1*, *pTAA1::TAA1-GFP*, *pWUS::GUS*, and *trn2-1* plants were previously described^9,13,17,20,24,48,51,56^. For plant materials at the mature flower stage, seeds were sown on vermiculite and Metro-Mix. For antibiotic selection, seeds were sown on half-strength Murashige and Skoog plates. All plants were grown at 22 °C under 24-h light conditions.

### Comparison of transcriptome datasets

Four available transcriptome datasets were used to identify genes that are regulated by the AG and CRC^6,17,34^. Genotypes and data sources are in Supplementary Table 1. Published lists of differentially expressed genes were used for *ag-11 ap2-35* and *ag-11 ap2-43* mutants^6^. For the *ag-12* mutant, raw data were downloaded from TAIR and analyzed using BIOCONDUCTOR in R. A filter of > 1.5-fold change was set. Venn diagrams were generated by VENNY v.2.1.0 (http://bioinfogp.cnb.csic.es/tools/venny/index.html). MeV (http://mev.tm4.org/#/welcome) was used to generate a heatmap and perform k-mean clustering. A gene ontology (GO) term enrichment analysis was conducted using the agriGO web-based tool and database^35^ (http://bioinfo.cau.edu.cn/agriGO/). The TreeMap view and interactive graph view of GO terms were generated with REVIGO (http://revigo.irb.hr/) after minimizing redundantly enriched GO terms^68^.

### Mutant phenotyping

To minimize environmental differences in growth chambers, the plants used for phenotyping were grown side-by-side at the same density in pots. The first five fruits from six different plants were observed for each genotype. A Chi-Square test was conducted to evaluate the statistical significance of the observed data.

### Chemical treatment

For dex treatment, the inflorescence containing *gCRC-GR*, *35S::AG-GR*, or *35S::AP1-GR* were sprayed once with 10 µM dex and 0.015% Silwet when plant heights were 4–8 cm. The same amount of solvent and Silwet was used as a control. For expression analysis, RNA from *crc-1 gCRC-GR*, *ag-1 35S::AG-GR*, L*er*, *gCRC-GR, 35S::AG-GR*, and *gCRC-GR 35S::AG-GR* was extracted four hours after dex treatment. For ChIP, *ap1 cal-1 35S::AP1-GR gCRC-myc* was fixed for four days after dex treatment when most floral buds were in developmental stage 6. For auxin treatment, a 30 µL solution of 10 µM NAA (Wako) and 0.015% Silwet was dropped onto inflorescences. Seven days after treatment, phenotype was observed.

### Plasmid construction and plant transformation

For *gCRC-GFP* construction, a genomic fragment covering 3492-bp upstream of the CRC translation start site and 1204-bp of the coding region was amplified from genomic DNA extracted from L*er* and cloned into the binary vector pGreen0311 (for mGFP). To construct *pYUC4::GUS*, the 3.7 kbp *YUC4* promoter was amplified using genomic DNA from L*er* as the PCR template and sub-cloned into pENTR/D-TOPO (Thermo Fisher Scientific) according to the manufacturer’s protocol (Thermo Fisher Scientific). Site-directed mutagenesis was conducted using a PrimeSTAR Mutagenesis Basal Kit (Takara) to obtain *pYUC4m*-pENTR/D-TOPO. The resulting *pYUC4* fragments were Gateway-cloned into *pBGWFS7* with the LR reaction. To construct *pCHR11::GUS* and *pCHR17::GUS*, the 1 kbp *CHR11* promoter and 0.5 kbp *CHR17* promoter were amplified using genomic DNA from Col as the PCR template and sub-cloned into pENTR/D-TOPO according to manufacturer’s protocol (Thermo Fisher Scientific). The resulting *pCHR11* and *pCHR17* fragments were Gateway-cloned into *pBGWFS7* vector by the LR reaction. To generate *35S::GFP-CHR11*, the 3.1 kbp *CHR11* cDNA was amplified using cDNA synthesized from Col as the PCR template and was sub-cloned into pENTR/D-TOPO according to the manufacturer’s protocol (Thermo Fisher Scientific). The resulting *CHR11* fragment was Gateway-cloned into the *pGWB6* vector by the LR reaction. To generate *pCRC::YUC4*, the 3.5 kbp *CRC* promoter and *YUC4* coding sequence were amplified using genomic DNA and cDNA from L*er* as PCR templates and the products were sub-cloned into different entry vectors. Whole functional fragments were cloned into the *pBGW* destination vector.

To generate deletion vectors, gRNAs fused with *Arabidopsis* tRNA-Gly gene were amplified using the artificial DNA fragment (gBlock Gene Fragments) as the PCR template (Supplementary Data 3; Integrated DNA Technology). The resulting PCR products harboring two independent gRNA sequences were cloned into the AarI-digested pKI1.1R vector^69^ using In-Fusion reaction according to the manufacturer’s protocol (Clontech). All insertion sequences were confirmed with an ABI 3130 × 1 Sequencer (ABI) and 3130 × 1 series Data Collection 4 software (ABI). All primers used for cloning and genotyping are listed in Supplementary Data 3.

For plant transformations, inflorescences were dipped in *Agrobacterium tumefaciens* containing plasmids for 1 min and incubated for 1 day in humid conditions at room temperature. T1 seeds were collected and screened for antibiotic resistance. More than 20 T1 plants were screened, and representative lines were chosen for further characterization.

### qRT-PCR

Using an RNeasy Plant Mini Kit (Qiagen), total RNA was extracted from *Arabidopsis* floral bud clusters up to stage 7 and stage 10 for *YUC4/AG/CRC* and *WUS* expression analysis, respectively. Genomic DNA was digested using an RNase-free DNase Set (Qiagen) prior to cDNA synthesis. Reverse transcription was performed using the PrimeScript RT Master Mix (Takara) according to manufacturer’s protocol. The concentration of cDNA was determined with a Light Cycler 480 (Roche) and the Light Cycler 480 release 1.5.1.62 SP software (Roche) using the FastStart DNA Essential DNA Green Master (Roche). The detected signal was normalized to that of the internal control gene, *EIF4* (*At3g13290*). The qRT-PCR experiments were repeated three times with more than four technical replicates. The values are represented as the means ± SEMs. Two-tailed Student’s *t*-test was conducted to evaluate the statistical significance of the observed data. Primers used for qRT-PCR are listed in Supplementary Data 3.

### Confocal microscopy

For confocal microscope observations, plants were sown on vermiculite and Metro-Mix until plant height was 2–4 cm. The apical 0.5 cm of the inflorescence tips was removed with tweezers, immediately embedded into 5% agar (Difco), and sliced with a Liner Slicer PRO7 vibratome (Dosaka). The resulting plant sections were placed on glass slides, mounted on a drop of water, and immediately observed under a FV 1000 (Leica) microscope with FV10-ASW software (Leica). More than 10 plants were observed, and representative images are shown.

### Phylogenetic shadowing

The 5′ intergenic sequence of *YUC4* from *Arabidopsis thaliana* was downloaded from TAIR. The *YUC4* promoter region was used as a query to obtain the *YUC4* promoter sequence in the other seven *Brassicaceae* species using NCBI blastn. The resulting eight *YUC4* promoter sequences were aligned by mVISTA (http://genome.lbl.gov/vista/mvista/submit.shtml), and the promoter sequences were aligned using CLUSTALW (http://www.genome.jp/tools/clustalw/). Conserved *cis*-elements were visualized by WebLogo (http://weblogo.berkeley.edu).

### ChIP

For the CRC ChIP in the *ap1 cal* double mutant background, 0.3 g of inflorescences was used. The *ap1-1 cal-1 35S::AP1-GR gCRC-myc* inflorescences were treated once with 10 µM DEX and harvested at 4 days after treatment, which correspond to stage 6–7. For the Pol II, AG, and CHR11-GFP ChIPs, 0.6 g of inflorescences was used. Inflorescences were fixed in 1% formaldehyde for fifteen minutes. To extract chromatin, fixed frozen tissues were homogenized with a mortar and pestle. The resulting chromatin was sonicated to produce DNA fragments shorter than 500 bp and washed twice. IP was performed overnight. The myc (9E10; Santa Cruz), Pol II (4E8; Abcam), AG^70^, and GFP (A6455; Thermo Fisher Scientific) antibodies were used. After a wash with low and high salt buffer^71^, reverse cross-linking was conducted. The resulting DNA was purified using a Qiaquick DNA Purification Kit (Qiagen). DNA was quantified with a Light Cycler 480 (Roche) and Light Cycler 480 release 1.5.1.62 SP software (Roche) using FastStart DNA Essential DNA Green Master (Roche). The ChIP experiments were repeated three times with more than three technical replicates. The values are represented as the means ± SEMs. Two-tailed Student’s *t*-test was conducted to evaluate the statistical significance of the observed data. *TA3* was used as the internal control. Primers used in the ChIP experiment are listed in Supplementary Data 3.

### Scanning electron microscopy

Fruits were fixed in FAA overnight at room temperature and dehydrated with an ethanol and acetone series. Critical point drying with liquid CO_2_ and a gold coating were performed using EM CPD300 (Leica) and E-1010 (Hitachi), respectively. The tissues were observed under an S-4700 microscope (Hitachi) with an accelerating voltage of 15 kV. More than 10 fruits were observed under SEM, and representative images are shown.

### FAIRE

For FAIRE, 0.3 g of inflorescences was fixed in 1% formaldehyde for eight minutes. To extract chromatin, fixed frozen tissues were homogenized with a mortar and pestle. The resulting chromatin was sonicated to produce DNA fragments shorter than 500 bp and washed five times with wash buffer. To isolate nucleosome-depleted regions, a phenol–chloroform DNA extraction was performed^61,72^. After purification of DNA by Qiaquick DNA purification, DNA was quantified with a Light Cycler 480 (Roche) and the Light Cycler 480 release 1.5.1.62 SP software (Roche) using FastStart DNA essential DNA Green Master (Roche). FAIRE experiments were repeated twice with four technical replicates and the combined data are shown. Two-tailed Student’s *t*-test was conducted to evaluate the statistical significance of the observed data. Primers used for FAIRE are listed in Supplementary Data 3.

### Yeast two-hybrid assay

The *AG*, *CRC*, *ARF19*, and *IAA14* cDNA fragments were amplified using PrimeSTAR GXL DNA Polymerase (Takara). Complementary DNA was prepared from RNA extracted from *Arabidopsis* Col and used as the PCR template. The resulting DNA fragments were introduced into pENTR/D-TOPO according to the manufacturer’s protocol (Thermo Fisher Scientific). Then, cDNA fragments were Gateway-cloned into the *pDEST* vector (Thermo Fisher Scientific) by the LR reaction. Bait and pray constructs were co-transformed into the MaV203 yeast strain. Selection for double transformants was performed on –Trp–Leu SD media. A survival test was conducted using –Trp–Leu–His SD media. Empty constructs and ARF19 and IAA14 interaction were examined as negative and positive controls, respectively^73^.

### Auxin measurement

For auxin measurements, floral buds were harvested when plant height reached 4–8 cm. Flowers older than stage 8 were manually removed using a dissecting microscope. One-hundred milligrams of floral bud clusters was used. Auxin was extracted and semi-purified^74^. IAA was quantified with an ultra-high performance liquid chromatography (UHPLC)-electrospray interface (ESI) and quadrupole-orbitrap mass spectrometer (UHPLC/Q-Exactive™; Thermo Fisher Scientific) with an ODS column^75^ (AQUITY UPLC HSS T3, 1.8 µm, 2.1 × 100 mm; Waters). Auxin measurements were repeated twice, and the combined data were shown.

### Transmission electron microscopy

For TEM, inflorescences were harvested when plants were 4–8 cm tall. Tissues were fixed in 2% paraformaldehyde and 1.25% glutaraldehyde in 0.05 M PB buffer for five hours at 4 °C, washed five times with 0.05 M PB buffer for 10 min at 4 °C, and fixed with OsO_4_ buffer at 4 °C overnight. The resulting tissues were washed with 8% sucrose in water for two hours at 4 °C, dehydrated with an ethanol series, and infiltrated with Eponate 812 by incubating the samples at room temperature for several hours to overnight in increasing concentrations of resin. Then, the resin was polymerized in an oven at 60 °C for 48 h. Resin-embedded samples were sectioned to 70 nm widths with a diamond knife on a ultramicrotome. Sections were collected on a 0.5% formvar coated slot grid. Grids were post-stained for 5 min with 2% aqueous uranyl acetate and for 10 min with Reynolds lead citrate. Images were taken using a H-7100 TEM (Hitachi).

### Sectioning

Fruits were fixed in FAA overnight at room temperature, dehydrated with an ethanol series, embedded in Technovit 7100 resin (Heraeus), and polymerized at room temperature overnight. Sections were made with a RM2255 microtome (Leica), extended onto slide glasses with a drop of water, and dried on a hot plate at 40 °C. After background staining with toluidine blue, sections were observed under an Axio Scope A1 microscope (ZEISS) equipped with an AxioCam ERc 5 s camera (ZEISS) and analyzed using ZEN2 software (ZEISS). More than five sections were observed, and representative images were shown.

### GUS staining

Inflorescences of 4–8 cm in height were fixed in acetone for 20 min at room temperature, rinsed with water, and stained with GUS staining solution. Sectioning was performed as stated above starting with dehydration in 70% ethanol through Technovit sectioning. For background staining, 0.05 % neutral red was used. Sections were observed under an Axio Scope A1 microscope (ZEISS) equipped with an AxioCam ERc 5 s camera (ZEISS) and analyzed using the ZEN2 software (ZEISS). To avoid positional effects of the transgene, exactly the same line was used in the wild type and mutants, generated by crossing. More than five sections were observed, and representative images are shown.

### Immunofluorescence localization

Sectioning was performed as described in the sectioning section. For fixation, cacodylic acid was used instead of FAA^76^. All monoclonal antibodies (JIM7, LM5, and LM15) were obtained from Plantprobes (http://www.plantprobes.net/index.php) and used at a dilution of 1/10 (v/v) as primary antibodies. 2/10 (v/v) was used for Supplementary Fig. 16f and g. Alexa Fluor 488 (Thermo Fisher Scientific) was used for labeling. Immunolocalization experiments were performed at least three times per treatment using three independent sections, and representative images are shown.

### In situ hybridization

Inflorescences of 4–8 cm in height were fixed in FAA at room temperature, dehydrated in a graded ethanol series, replaced with xylene and embedded in Paraplast (Sigma). Then, 8 µm Palaffin sections were made using a RM2255 microtome (Leica). Sections were dewaxed and rehydrated. Hybridization was conducted in a humid chamber at 55 °C overnight. The resulting sections were washed with SSC. Antibody binding and detection were performed using a DIG Labeling Kit (Roche). The 907-bp *YUC4* probe was used to detect *YUC4* mRNA. Primers used in the in situ hybridization are listed in Supplementary Data 3.

## Data Availability

Data supporting the findings of this study are available from the corresponding author upon reasonable request. A reporting summary for this Article is available as a Supplementary Information file.

## Supplementary information

Supplementary Information
Description of Additional Supplementary Files
Supplementary Data 1
Supplementary Data 2
Supplementary Data 3
Peer Review File
Reporting Summary
