# Supplementary material for: Chromatin-mediated feed-forward auxin biosynthesis in floral meristem determinacy
Source: Nat Commun. 2018 Dec 11;9:5290. doi: 10.1038/s41467-018-07763-0 (PMC6289996; doi:10.1038/s41467-018-07763-0)
Supplement: Supplementary file 1 — Supplementary Information [file 41467_2018_7763_MOESM1_ESM.pdf]

Supplementary Materials

Chromatin-mediated feed-forward auxin biosynthesis in floral meristem determinacy
Yamaguchi et al.

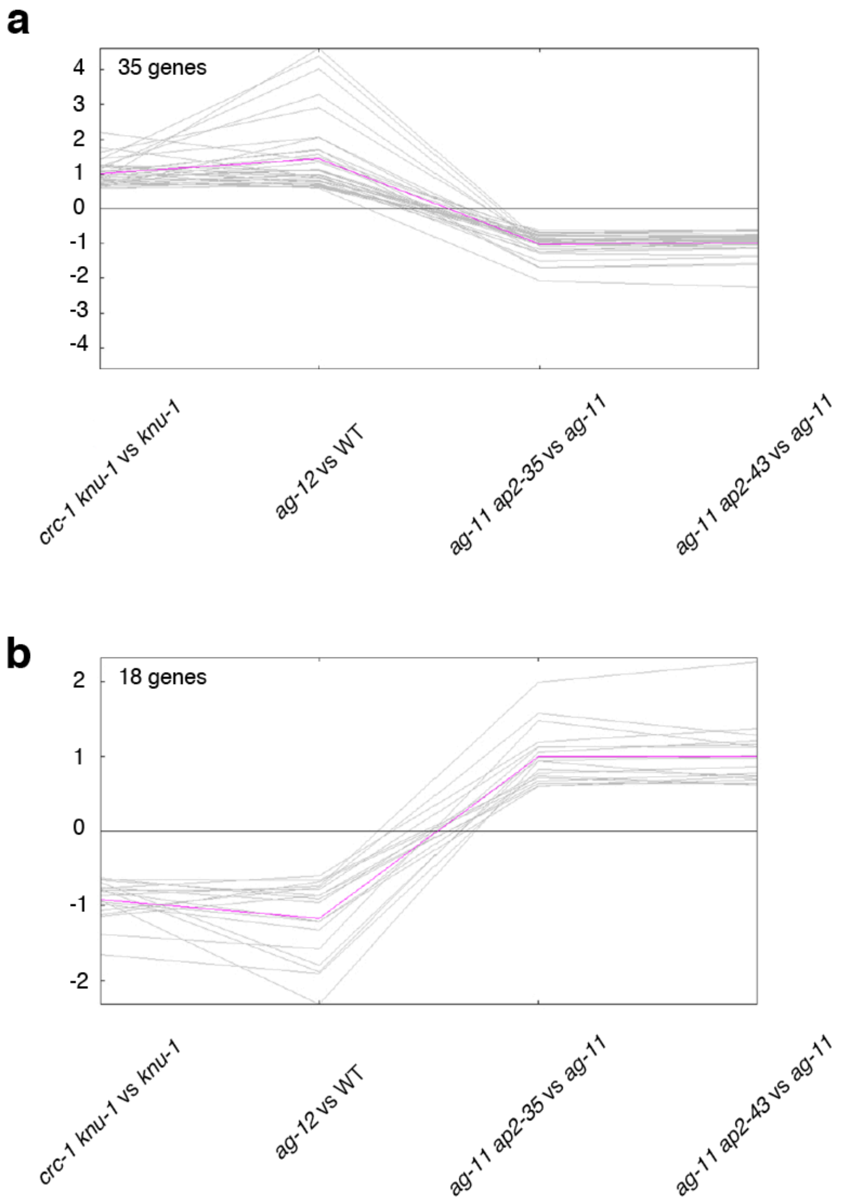

Supplementary Figure 1 Gene expression changes of 53 genes downstream of AG and CRC. **a** Graphs display $\log_2$ expression changes of the 53 targets based on four public transcriptome datasets. One cluster contains the 35 genes that are upregulated in *crc−1 knu−1* compared to *knu−1*. **b** The other cluster contains the 18 genes that are downregulated in *crc−1 knu−1* compared to *knu−1*.

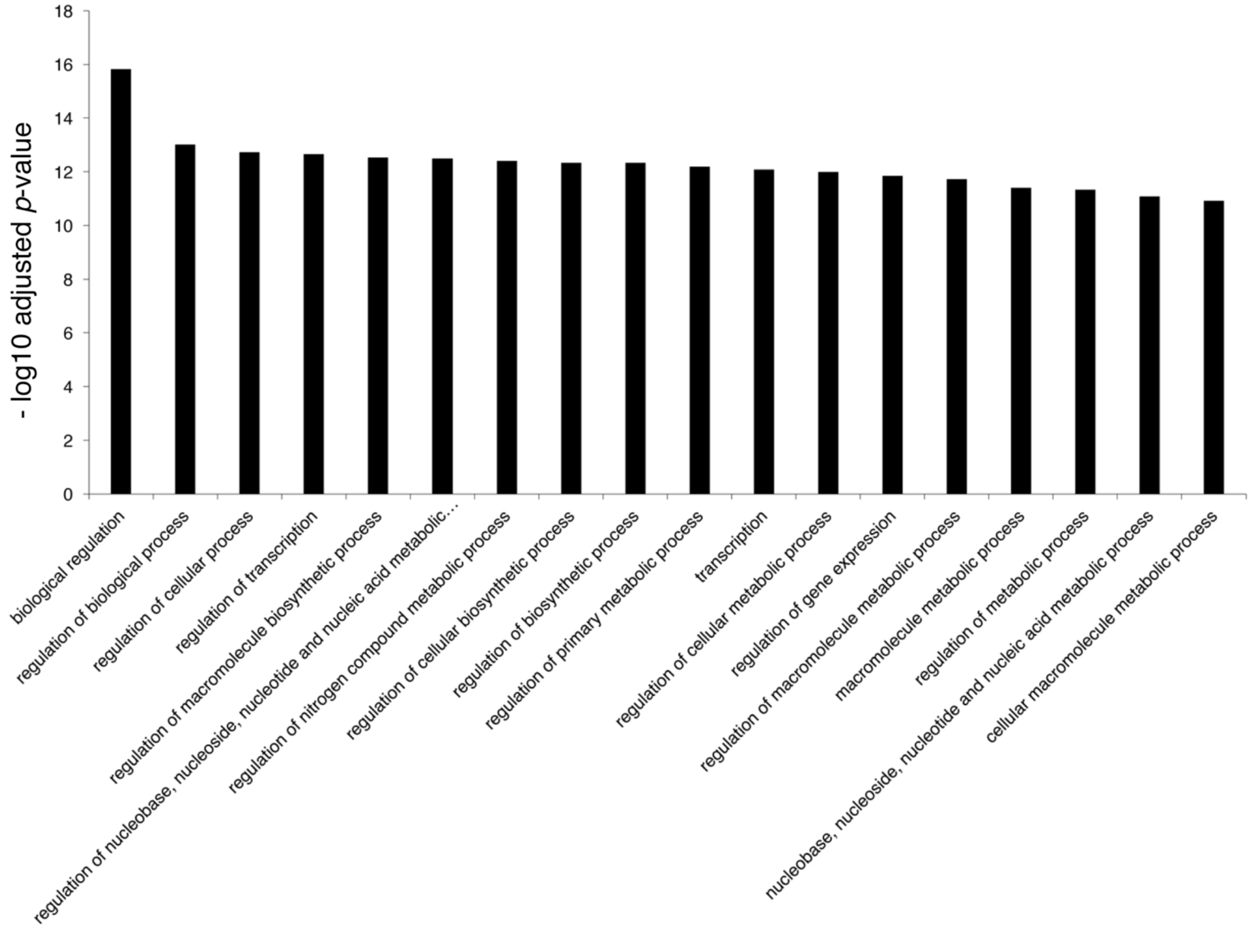

Supplementary Figure 2 GO term enrichment analysis of the 53 genes. Graphs display $-\log_{10}$ adjusted *p*-values of the 53 targets based on agriGO followed by REVIGO. The false discovery rate (FDR) was lower than 1.7×10^{-10}.

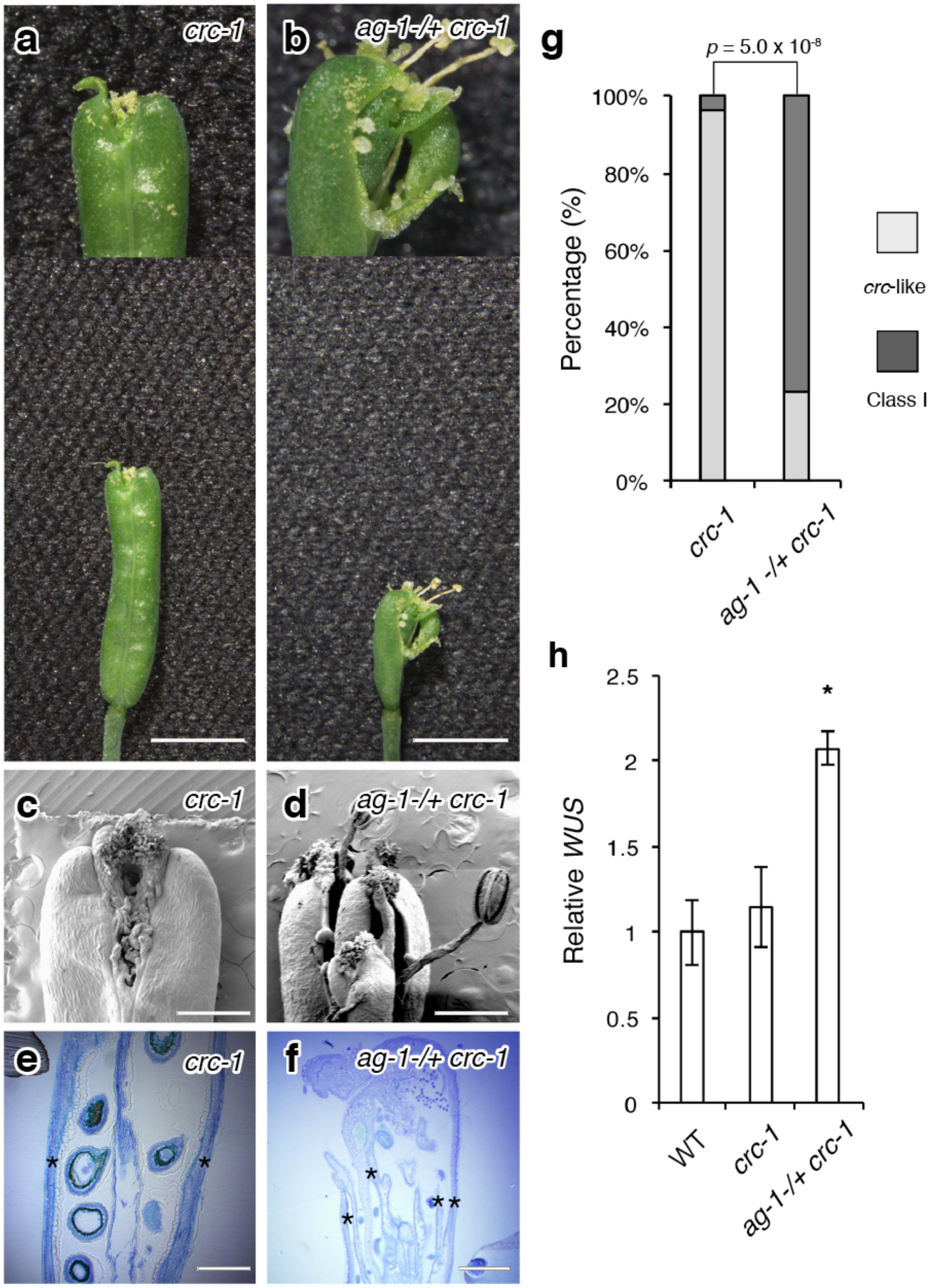

Supplementary Figure 3 Indeterminate phenotype of *ag–1 –/+ crc–1*. **a**, **b** Morphology of *crc–1* (**a**) and *ag–1 –/+ crc–1* (**b**) fruits. Above: Close–up views of fruit tips. Below: Whole fruits. Arrowheads indicate stigma structures. **c**, **d** Scanning electron micrograph of *crc–1* (**c**) and *ag–1 –/+ crc–1* (**d**) fruits. **e**, **f** Longitudinal section of *crc–1* (**e**) and *ag–1 –/+ crc–1* (**f**) fruits. Asterisks indicate carpels. **g** Quantification of the mutant phenotype. *p*–values were calculated with a Chi–Square test. **h** mRNA abundance of the stem cell marker *WUS* in wild–type, *crc–1*, and *ag–1–/+ crc–1* flowers. The values are represented as the means ± SEMs. *p*–values were calculated with a Student's *t*–test. Bars = 1 cm in **a**, **b**; 1 mm in **c**, **d**; 500 μm in **e**, **f**.

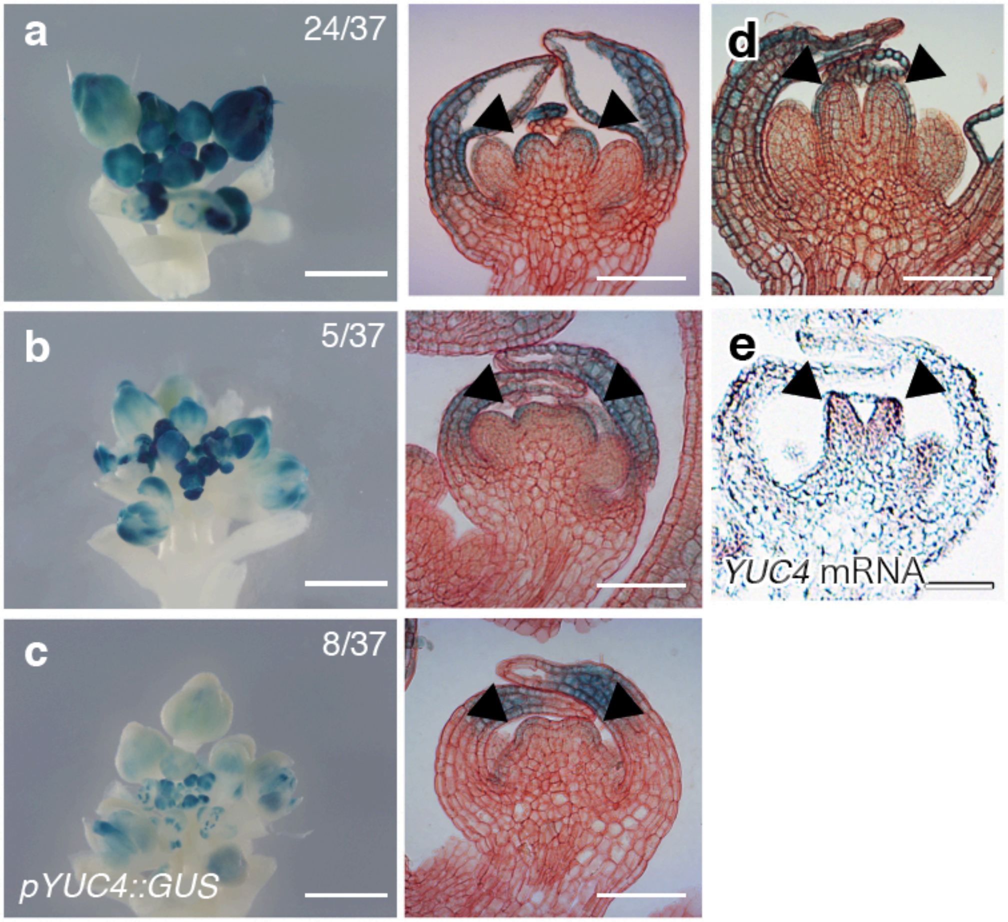

Supplementary Figure 4 *YUC4* expression in floral buds. **a–c** GUS staining of *pYUC4::GUS* in the inflorescences (left) and stage 6 floral buds (right). Independent T1 lines were categorized in three difference classes depending on expression levels: lines with strong (**a**), intermediate (**b**), and weak (**c**) signals. Specifically, 24, 5, and 8 out of 37 lines showed strong, intermediate, and weak expression, respectively. The representative line from the strong category were shown. **d** GUS staining of *pYUC4::GUS* in the stage 7 floral buds. **e** *YUC4* expression pattern, as determined by *in situ* hybridization. Arrowheads indicate abaxial side of epidermal cells in carpels at stage 6. Bars = 3 mm in **a–c** (left); 50 μm in **a–c** (right), **d, e**.

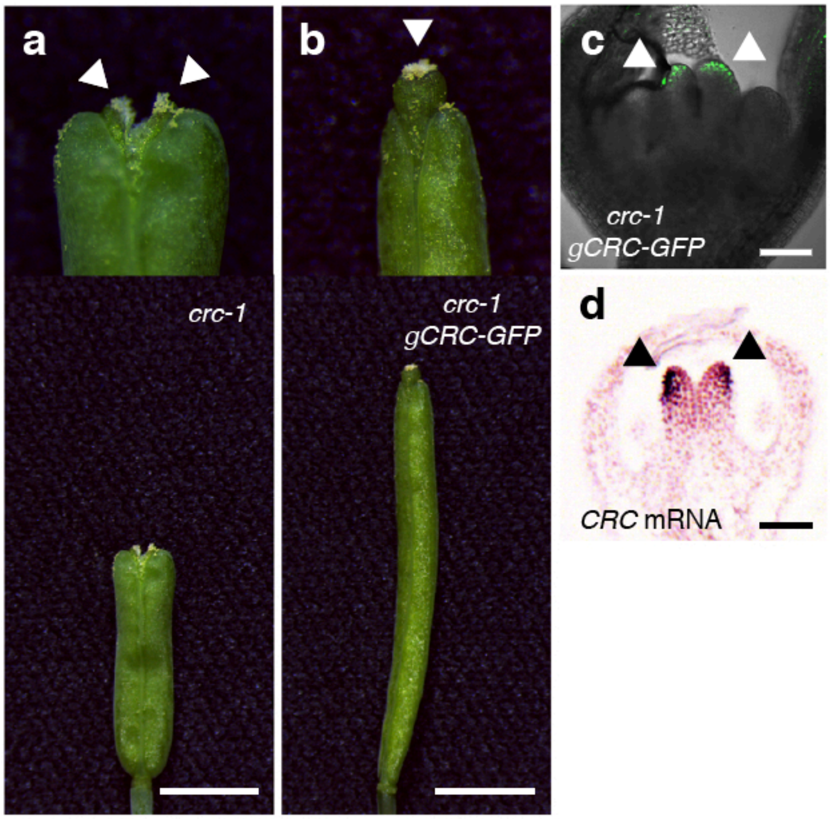

Supplementary Figure 5 Expression of g*CRC-GFP* and *CRC* mRNA. **a**, **b** Phenotypic rescue of the *crc-1* mutant phenotype by *gCRC-GFP* expression. Morphology of *crc-1* (**a**) and *crc-1 gCRC-GFP* (**b**) plants. Top: Close-up views of fruit tips. Bottom: Whole fruits. Arrowheads indicate stigma structures. **c** *gCRC-GFP* expression in wild-type stage 6 floral buds. **d** *In situ* hybridization of *CRC* mRNA in wild-type stage 6 floral buds. Arrowheads indicate the abaxial side of carpel primordia. Bars = 5 mm in **a**, **b**; 50 μm in **c**, **d**.

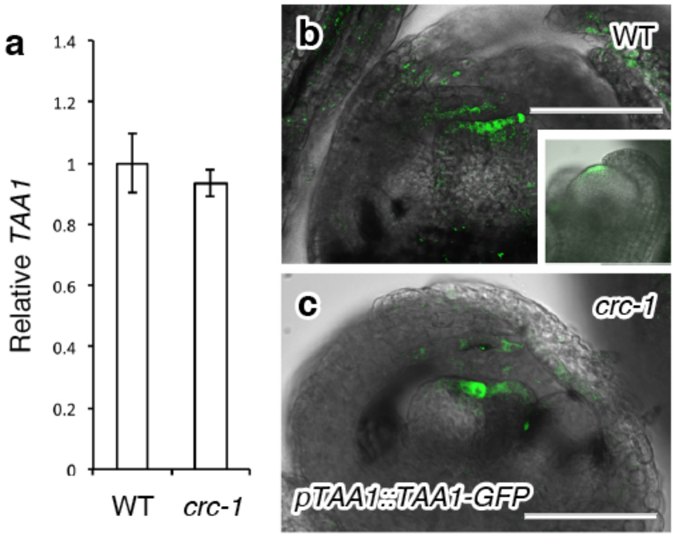

Supplementary Figure 6 *TAA1* expression in the *crc-1* mutant. **a** *TAA1* expression in wild-type, and *crc-1*. The values are represented as the means ± SEMs. **b**, **c** TAA1-GFP in longitudinal sections of wild-type (**b**), and *crc-1* (**c**) stage 6 floral buds. Inset was TAA1-GFP expression in stage 4 floral buds. Bars = 50 μm in **b**, **c**.

```
4TarvenseYUC4:58907-59887        --------TC-TAATATCATGTACTATGATGAGACAAACTGAGGGCAAATTCAAGCCTGA
BnapusYUC41:12408-12873          TGTGTGGTCATGTAACTATGATCTATGATGAGACAAAATGAGGGAGAAATTCAAGCCTGA
BrapaYUC4:18227-18692            TGTGTGGTCATGTAACTATGATCTATGATGAGACAAAATGAGGGAGAAATTCAAGCCTGA
LalabamicaYUC4:10757-11228       AATGTGGTCATGTAACTATGTTGA--GACAAATCGAGAGTGAGGGCAAATTCAAGCCTGA
AthaYUC4:1570-2042               AGTGTGGTCATGTAACTATGGTGA--GAAAAATTGAGGTTGAGGGCAAATTCAAGCCTGA
AlyraYUC4:240239-240713          AGTGTGGTCATGTAACTATGGTTA--GACAAATTGAGGTTGAGGGCAAATTCATGCCTGA
CrubellaYUC4:86652-87138         AGTGTGGTCATGTAACTATGGTGA--GACAAATTGAGGCTGAGGGCAAATTCAAGCCTGA
CsativaYUC4:41129-41606          AGTGTGGTCATGTAACTAT---------GGTATTGAGGTTGAGGGCAAATTCAAGCCTGA
                                              *  *                  *      **  ******* ******

TarvenseYUC4:58907-59887         CAGAAAAATTCCAAAGAGCATTGGGTCGTAAAAT--------GCAGAAGGAGAA-AATTA
BnapusYUC41:12408-12873          CAGAAAAATTCCAAAGAGCACTGAGTCGGGAGGAT-------ACAGAATGAGAA-ATTTG
BrapaYUC4:18227-18692            CAGAAAAATTCCAAAGAGCACTGAGTCGGGAGGAT-------GCAGAATGAGAA-AATTG
LalabamicaYUC4:10757-11228       CAGAAAAATTCCAAAGGCATTGTCTCGGGTTAATG--------CATAAGAAGAAAATAGA
AthaYUC4:1570-2042               CAGAAAAGTTCCAAAGAGC--------ATTGAGTCGGATAATGCAGAAGGAGAA-ATTGG
AlyraYUC4:240239-240713          CAGAGAAATTCCAAAGAGC--------AGTGAGTAGGATAATGCAGAAGGAGAA-AATGG
CrubellaYUC4:86652-87138         CAGAAAAATTCCAAAGAGAAT------ACTGAGTCGGATAATGCAGAAGGAGAA-AATGG
CsativaYUC4:41129-41606          CAGAAAAATTCCAAAGAGCATTGAGTCGGAATGTCGGATAATGCAGAAGGAGAA-AATGG4
                                 **** ** ********                           ** **  **** *

TarvenseYUC4:58907-59887         TAGAAGAGGGCAGAGG----AAGATGACGCTTTTCTGGGACCCAAAATCTTTTTTCTACT
BnapusYUC41:12408-12873          AAGAAGATGCCAGAGGAAGATATATGACGCTTGTGTAGGACCTAAAATCGTTTGTCTACA
BrapaYUC4:18227-18692            AAGAAGATGCCAGAGGAAGATATATGACGCTTGTGTAGGACCTAAAATCTTTTGTCTACA
LalabamicaYUC4:10757-11228       AATAAGAGTCCAAAGG----AAGATGATGTTT--TTGTAGGACATGATACTTTTGTCTGC
AthaYUC4:1570-2042               AAGAAGAGGCCAGAGC----AAGATGACGTTTTCATGGGACCCTAAATTCTTTTGTCTAC
AlyraYUC4:240239-240713          AAGAAGATGCCAGAGG----AAGATGACGTTTTTATGGGACCCTAAACTCTTTTGTCTAC
CrubellaYUC4:86652-87138         AAGAAGAGGCCAGAGG----AAGATGACGTTTTTATGGGACCGTTAATTCTTTTGTCTAC
CsativaYUC4:41129-41606          TAGAAGAGGCCAGAGG----AAGATGACGTTTTTGAGGGACCCTAAATTCTTTTGTCTAC
                                  * ****   ** **      * **** * **              *   ***

TarvenseYUC4:58907-59887         GTGTAAATATTCATTTTCTTGTCATTATATCCAAAGTATATATATGAACATCTCTATTGT
BnapusYUC41:12408-12873          GTATATAATATTCTTGTTTTGCCAT-----------------------------------
BrapaYUC4:18227-18692            GTATATAATATTCTTGTTTTGCCAT-----------------------------------
LalabamicaYUC4:10757-11228       ATTGTAATATTCATATTTTTACCAT-----------------------------------
AthaYUC4:1570-2042               ATTGTAATATTCATATTTTTGCCAT-----------------------------------
AlyraYUC4:240239-240713          ATTGTAATATTCATATTTTTGCCAT-----------------------------------
CrubellaYUC4:86652-87138         ATTGTAATATTCATATTTTTGCCAT-----------------------------------
CsativaYUC4:41129-41606          ATTGTAATGTTCATATTTTTGCCAT-----------------------------------
                                  *    *   *  *  * **  ***

TarvenseYUC4:58907-59887         GATTTCTTAATGAGAATCTTATACTACAAGTAAATTAAAAATAAATAAAAGATTGAGAAA
BnapusYUC41:12408-12873          ------------------------------------------------------------
BrapaYUC4:18227-18692            ------------------------------------------------------------
LalabamicaYUC4:10757-11228       ------------------------------------------------------------
AthaYUC4:1570-2042               ------------------------------------------------------------
AlyraYUC4:240239-240713          ------------------------------------------------------------
CrubellaYUC4:86652-87138         ------------------------------------------------------------
CsativaYUC4:41129-41606          ------------------------------------------------------------

TarvenseYUC4:58907-59887         AGATGATAACCTATTCTCAATTTAAAGATTTTGAGATCCATGTGAAAATCCTTAACATAA
BnapusYUC41:12408-12873          ------------------------------------------------------------
BrapaYUC4:18227-18692            ------------------------------------------------------------
LalabamicaYUC4:10757-11228       ------------------------------------------------------------
AthaYUC4:1570-2042               ------------------------------------------------------------
AlyraYUC4:240239-240713          ------------------------------------------------------------
CrubellaYUC4:86652-87138         ------------------------------------------------------------
CsativaYUC4:41129-41606          ------------------------------------------------------------

TarvenseYUC4:58907-59887         GGCATATACAGTATCAATATAATCTATGAGTGTGTGTGAGTAGTTCATATTTCCTGAAAA
BnapusYUC41:12408-12873          ------------------------------------------------------------
BrapaYUC4:18227-18692            ------------------------------------------------------------
LalabamicaYUC4:10757-11228       ------------------------------------------------------------
AthaYUC4:1570-2042               ------------------------------------------------------------
AlyraYUC4:240239-240713          ------------------------------------------------------------
CrubellaYUC4:86652-87138         ------------------------------------------------------------
CsativaYUC4:41129-41606          ------------------------------------------------------------

TarvenseYUC4:58907-59887         TAGATCAAAAACACAATAATTTAGAATATTAATTAAAAATTGTATGTTGTAATATATATT
BnapusYUC41:12408-12873          ------------------------------------------------------------
BrapaYUC4:18227-18692            ------------------------------------------------------------
LalabamicaYUC4:10757-11228       ------------------------------------------------------------
AthaYUC4:1570-2042               ------------------------------------------------------------
AlyraYUC4:240239-240713          ------------------------------------------------------------
CrubellaYUC4:86652-87138         ------------------------------------------------------------
CsativaYUC4:41129-41606          ------------------------------------------------------------
```

```
TarvenseYUC4:58907-59887          TAGATCAAAAACACAATAATTTAGAATATTAATTAAAAATTGTATGTTGTAATATATATT
BnapusYUC41:12408-12873           ------------------------------------------------------------
BrapaYUC4:18227-18692             ------------------------------------------------------------
LalabamicaYUC4:10757-11228        ------------------------------------------------------------
AthaYUC4:1570-2042                ------------------------------------------------------------
AlyraYUC4:240239-240713           ------------------------------------------------------------
CrubellaYUC4:86652-87138          ------------------------------------------------------------
CsativaYUC4:41129-41606           ------------------------------------------------------------

TarvenseYUC4:58907-59887          TATCAAAGTCTGTTATGCATAAGTTTAAATTATATACTAATATCATTTTCAATTGGTATT
BnapusYUC41:12408-12873           ------------------------------------------------------------
BrapaYUC4:18227-18692             ------------------------------------------------------------
LalabamicaYUC4:10757-11228        ------------------------------------------------------------
AthaYUC4:1570-2042                ------------------------------------------------------------
AlyraYUC4:240239-240713           ------------------------------------------------------------
CrubellaYUC4:86652-87138          ------------------------------------------------------------
CsativaYUC4:41129-41606           ------------------------------------------------------------

TarvenseYUC4:58907-59887          AGTTAGATTTAATGCAATAATATTATATTATTATTTTAATTAATATTAAAATATAAA
BnapusYUC41:12408-12873           ------------------------------------------------------------
BrapaYUC4:18227-18692             ------------------------------------------------------------
LalabamicaYUC4:10757-11228        ------------------------------------------------------------
AthaYUC4:1570-2042                ------------------------------------------------------------
AlyraYUC4:240239-240713           ------------------------------------------------------------
CrubellaYUC4:86652-87138          ------------------------------------------------------------
CsativaYUC4:41129-41606           ------------------------------------------------------------

TarvenseYUC4:58907-59887          AATGTGAAATTTGAAAAGAAAAAAAATATTATCTGTATAATTATGATTTTCTCATATCTA
BnapusYUC41:12408-12873           ------------------------------------------------------------
BrapaYUC4:18227-18692             ------------------------------------------------------------
LalabamicaYUC4:10757-11228        ------------------------------------------------------------
AthaYUC4:1570-2042                ------------------------------------------------------------
AlyraYUC4:240239-240713           ------------------------------------------------------------
CrubellaYUC4:86652-87138          ------------------------------------------------------------
CsativaYUC4:41129-41606           ------------------------------------------------------------

TarvenseYUC4:58907-59887          TATGATACTAAAGTATATATAATATTAATAAGTATATTAATATTATATTAATCAGAGGCT
BnapusYUC41:12408-12873           ------------------------------------------------------------
BrapaYUC4:18227-18692             ------------------------------------------------------------
LalabamicaYUC4:10757-11228        ------------------------------------------------------------
AthaYUC4:1570-2042                ------------------------------------------------------------
AlyraYUC4:240239-240713           ------------------------------------------------------------
CrubellaYUC4:86652-87138          ------------------------------------------------------------
CsativaYUC4:41129-41606           ------------------------------------------------------------

TarvenseYUC4:58907-59887          CTAAGCATCTAACATTGCTAATATTGATTGATCTATCATCATCTATCCAAGATCCACAAT
BnapusYUC41:12408-12873           --------TATCGATAGTTTATACAGCATTGCTGATGTTGATCCATCCAAGATCCACAAT
BrapaYUC4:18227-18692             --------TATCGATAGTTTATACAGCATTGCTGATGTTGATCCATCCAAGATCCACAAT
LalabamicaYUC4:10757-11228        --------TATCGATAGTGTATACAAGATTGATGATGTTGATCCATCCAAGTTCCACAAT
AthaYUC4:1570-2042                --------TATCGATAGTGTAAACAAGATTGCTGATGGTGATCCATCCAAGGACCACAAT
AlyraYUC4:240239-240713           --------TATCGATAGTGTAAACAAGATTGCTGATGTTGATCCATCCAAGAACCACAAT
CrubellaYUC4:86652-87138          --------TATCGATAGTTTATACAAGATTGCTGATGTTGATCCATCCAAGAACCACAAT
CsativaYUC4:41129-41606           --------TATCGATAGTTTATACAAGATTGCTGATGTTGATCCATCCAAGAACCACAAT
                                               ** *   * *     *     **  * *** *******  *******

TarvenseYUC4:58907-59887          ATTCCTCGTCTAACCAAAGGGACCATCGATACACCTATGTAACATGCATATAAAGTAGAT
BnapusYUC41:12408-12873           ATTCCTCGTCTAACCAAAGGGACCATCAATACATGCATGTAAAGTAA-------------
BrapaYUC4:18227-18692             ATTCCTCGTCTAACCAAAGGGACCATCAATACATGCATGTAAAGTAG-------------
LalabamicaYUC4:10757-11228        ATTCCTCGTCTAACCAAAGGGACCATGATGAATACATTATATGTAACATGCATATAAAGT
AthaYUC4:1570-2042                ATTCCTCGTCTAACCAAAGGGACCATCAATACACTATGTAACAT-GCA----TATAGAGT
AlyraYUC4:240239-240713           ATTCCTCGTCTAACCAAAGGGACCATTGATACTCTATATATGTA-ACATGCGTATAAAGT
CrubellaYUC4:86652-87138          ATTCCTCGTCTAACCAAAGGGACCATCGATACACTATGTAACAT-GCATATGAACGAAGT
CsativaYUC4:41129-41606           ATTCCTCGTCTAACCAAAGGGACCATCGATACACTATGTAACGT-GCATATAGAGTAGT-
                                  **************************    *

TarvenseYUC4:58907-59887          TTAC---TAGTAGCTGATAC------------------ACTTGTATTTTTCTTCCAGCTT
BnapusYUC41:12408-12873           --ATTACTAGTAGCTGATACACTTGCATAG-----CTTGT----AATTTTCTCCTAGATT
BrapaYUC4:18227-18692             --ATTACTAGTAGCTGATACACTTGCATAG-----CTTGT----AATTTTCTCCTAGATT
LalabamicaYUC4:10757-11228        ATATTACTAGTAGCTGATACACTTGTAT------------------TTTTCTTCCAGCTT
AthaYUC4:1570-2042                AGATTAATAGTATAGCCGA---TACACTTGTATAACTTGTAT----TTTTCTTCCAGCTT
AlyraYUC4:240239-240713           AGATT--AATAGTAGCTGA---TACACTTGTATAACTTGTAT----TTTTCTTCCAGCTT
CrubellaYUC4:86652-87138          AGATTAGTAGTAGCTGATACACTTGTATTGTATAACTTGTATTTTTCTTCCAGCAGGCTT
CsativaYUC4:41129-41606           ---TTAATAGTAGCTGATACACTTGTATAACTT----GTATTTTTCTTACCAGCTAGCTT
                                          *         *                            *  *  *  * **
```

Supplementary Figure 7 DNA sequence alignment of the CRM1 region in the *YUC4* promoter. DNA sequence information for all eight species was obtained from GenBank. No potential YABBY binding sites were found in this region.

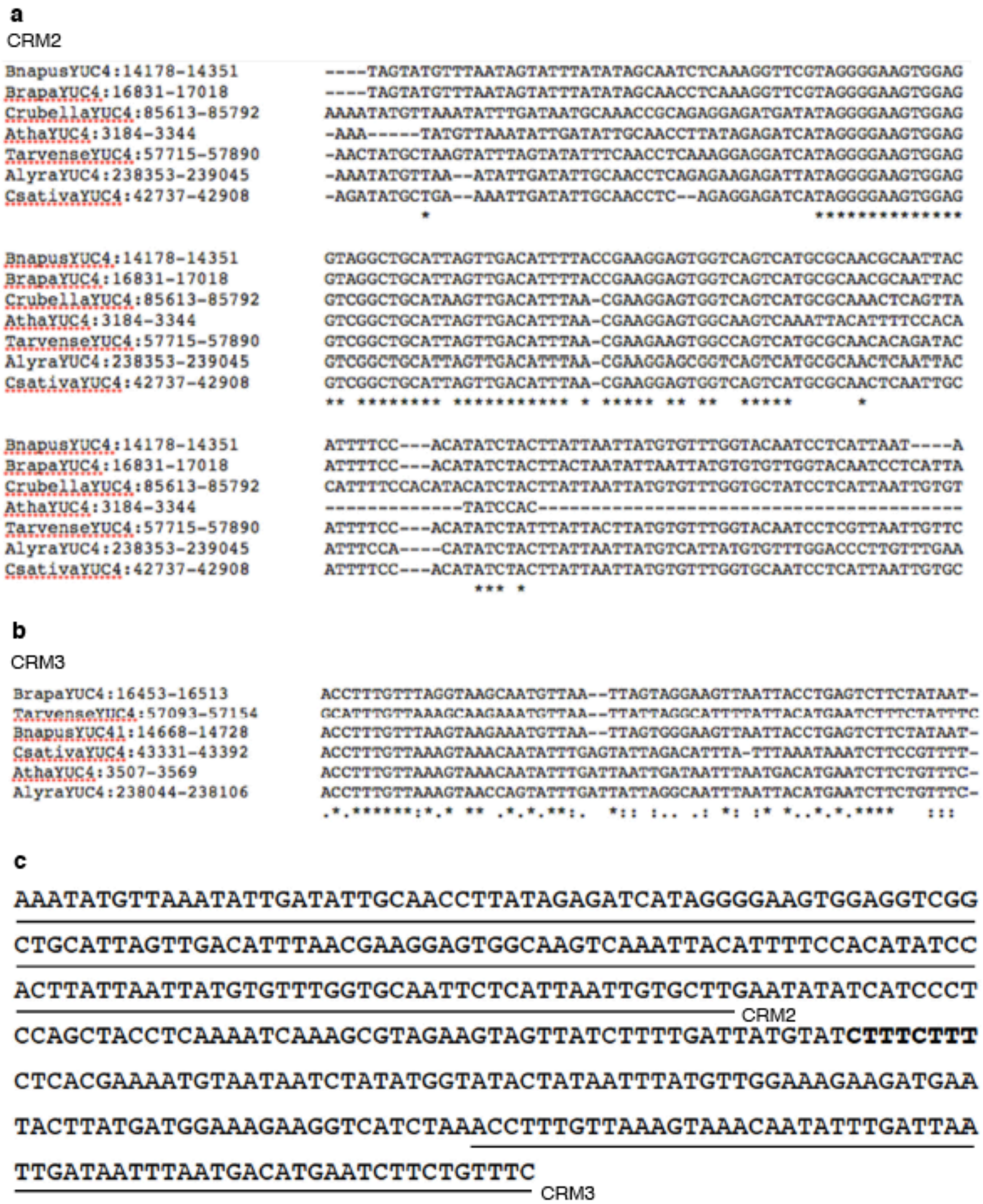

Supplementary Figure 8 DNA sequence alignment of the CRM2 and CRM3 regions in the *YUC4* promoter. **a**, **b** DNA sequence alignment of the CRM2 (**a**) and CRM3 regions (**b**). DNA sequence information for all eight spices was obtained from GenBank. **c** DNA sequence between CRM2 and CRM3 in the *Arabidopsis thaliana YUC4* promoter where a potential YABBY binding site (bold) was identified.

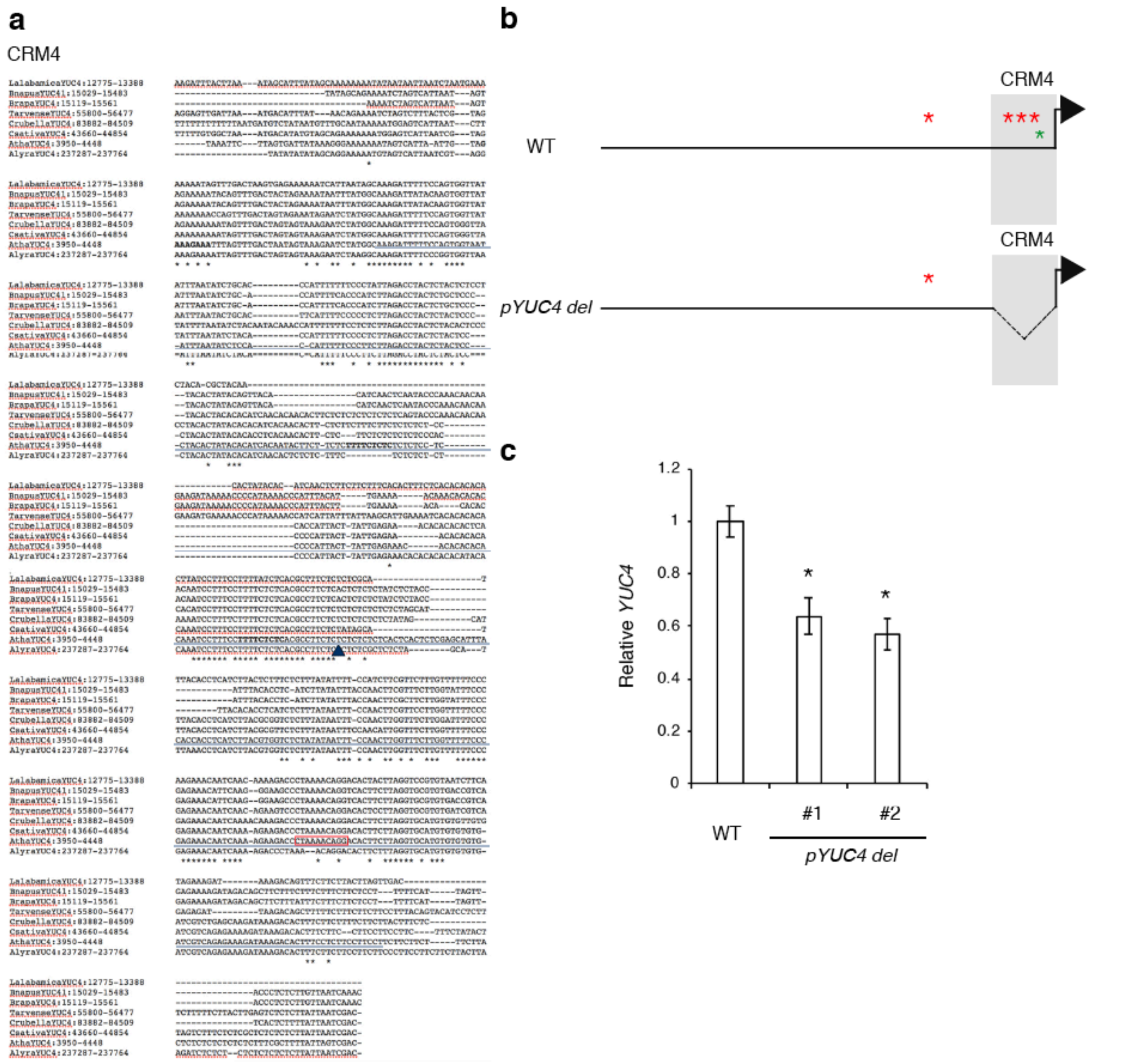

Supplementary Figure 9 DNA sequence alignment and functional analysis of the CRM4 region in the *YUC4* promoter. **a** DNA sequence alignment of the CRM4 region. DNA sequence information for all eight species was obtained from GenBank. Bold: three potential YABBY binding sites. Pale blue line: significant AG binding region. Blue triangle: position of the highest AG peak. Red square: possible CArG box. **b** Diagram of the CRM4 deletion by CRISPR/Cas. gRNA1 gRNA1, 5'-taaattcttagtgattataa-3'; gRNA2, 5'-gtaagaaagaagaagaagga-3'. **c** *YUC4* mRNA levels in the wild type and the CRM4 deleted lines. The values are represented as the means ± SEMs. *p*-values were calculated using a Student's *t*-test. *: $p < 0.05$ compared to the wild type.

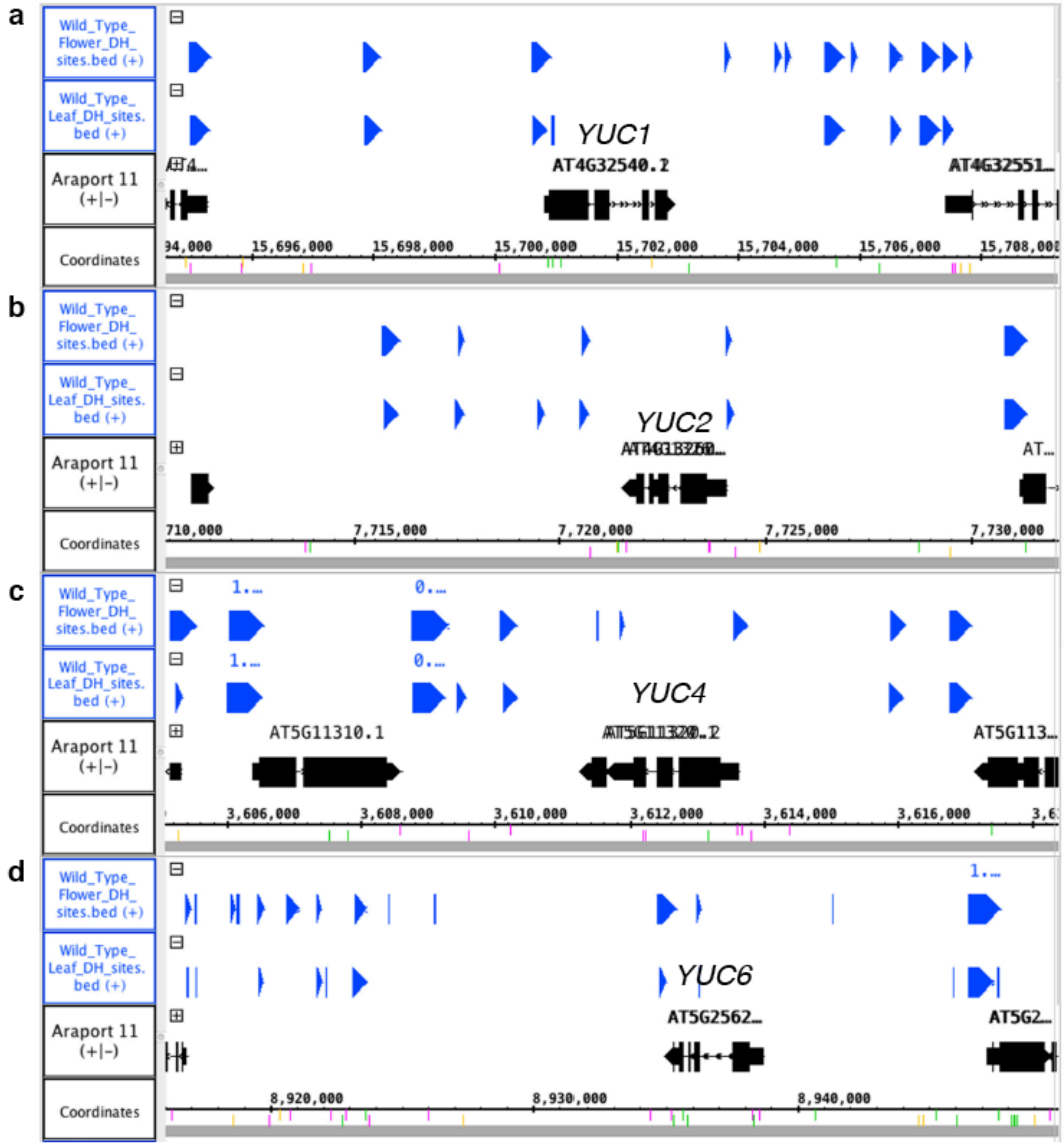

Supplementary Figure 10 Regulatory region of the *YUC* genes. **a**–**d** Identification of the YABBY-binding sites by a DNase I HS assay and sequences of potential YABBY-binding sites in the *YUC1* (**a**), *YUC2* (**b**), *YUC4* (**c**), and *YUC6* (**d**) genes. Pink, GA[GA]AGAAA; Green, CC[CA][TC]C[TA][CT]C; Yellow, CCCCAC. Flower-specific DNase I HS sites, which contain four potential YABBY-binding sites were specifically observed in the 5′ region of the *YUC4* promoter.

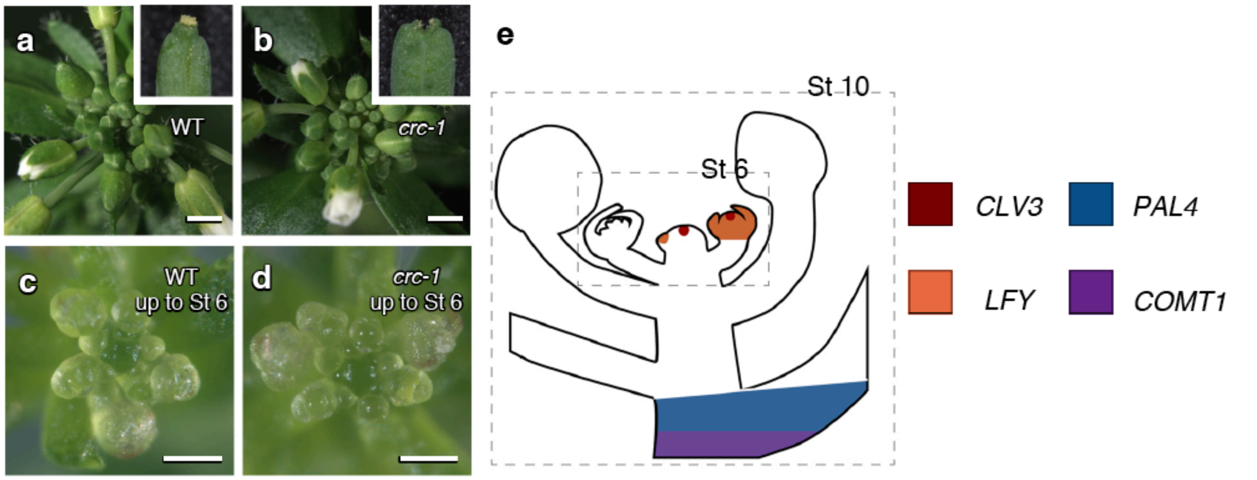

Supplementary Figure 11 Trimming of wild–type and *crc* inflorescences for auxin quantification. **a**, **b** Top view of wild–type (**a**), and *crc–1* (**b**) shoot apexes. Fruit tips were shown in insets. **c**, **d** Top view of wild–type (**c**) and *crc–1* (**d**) shoot apexes after removal of flowers from plants at stage 7 or later. Bars = 1 mm in **a**, **b**; 200 μm in **c**, **d**. **e** Schematic diagram of spatial *CLV3*, *LFY*, *PAL4*, and *COMT* expression patterns.

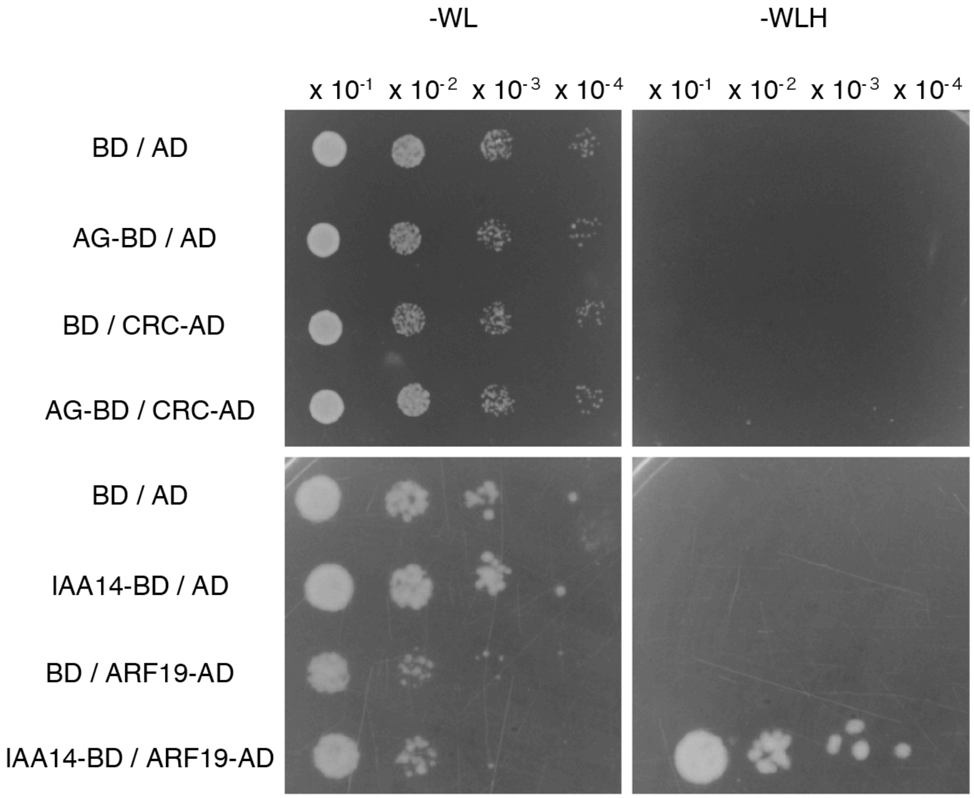

Supplementary Figure 12 Yeast–two hybrid test for an AG–CRC interaction. Left; Yeast grown under nonselective conditions (–WL). Right; Yeast grown under selective conditions (–WLH). Double transformants carrying both the *pDEST22* and *pDEST32* empty vectors were used as a negative control. A positive AUXIN RESPONSE FACTOR19 and IAA14 interaction was detected under our experimental conditions[1].

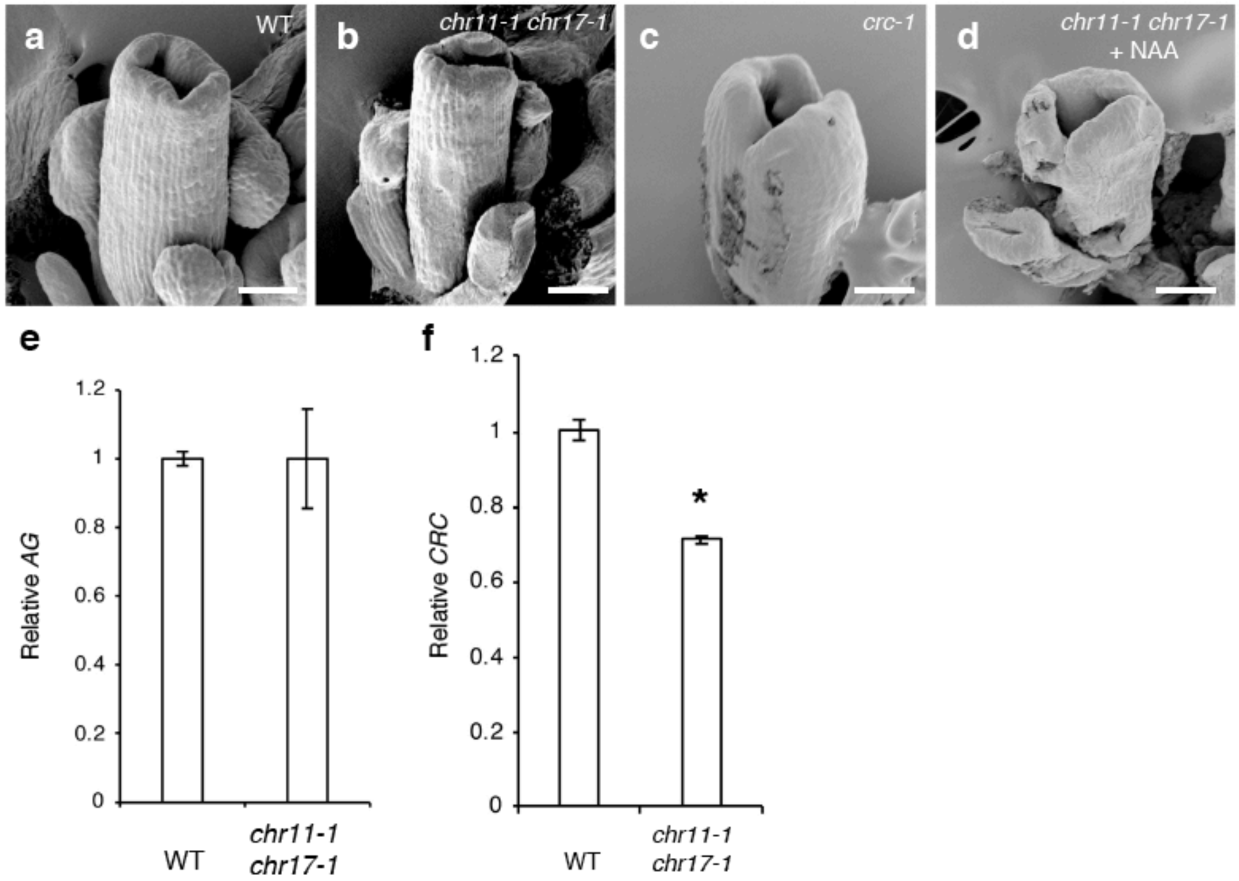

Supplementary Figure 13 Characterization of the *chr11-1 chr17-1* double mutants. **a**–**d** Scanning electron micrograph of the wild-type (**a**), *chr11-1 chr17-1* (**b**), *crc-1* (**c**), and auxin-treated *chr11-1 chr17-1* (**d**) gynoecia. **e**, **f** *AG* (**e**) and *CRC* (**f**) mRNA levels in the wild-type and *chr11-1 chr17-1* floral buds. The values are represented as the means ± SEMs. *p*-values were calculated using a Student's *t*-test. *: $p < 0.05$ compared to the wild type. Bars = 60 μm in **a**–**d**.

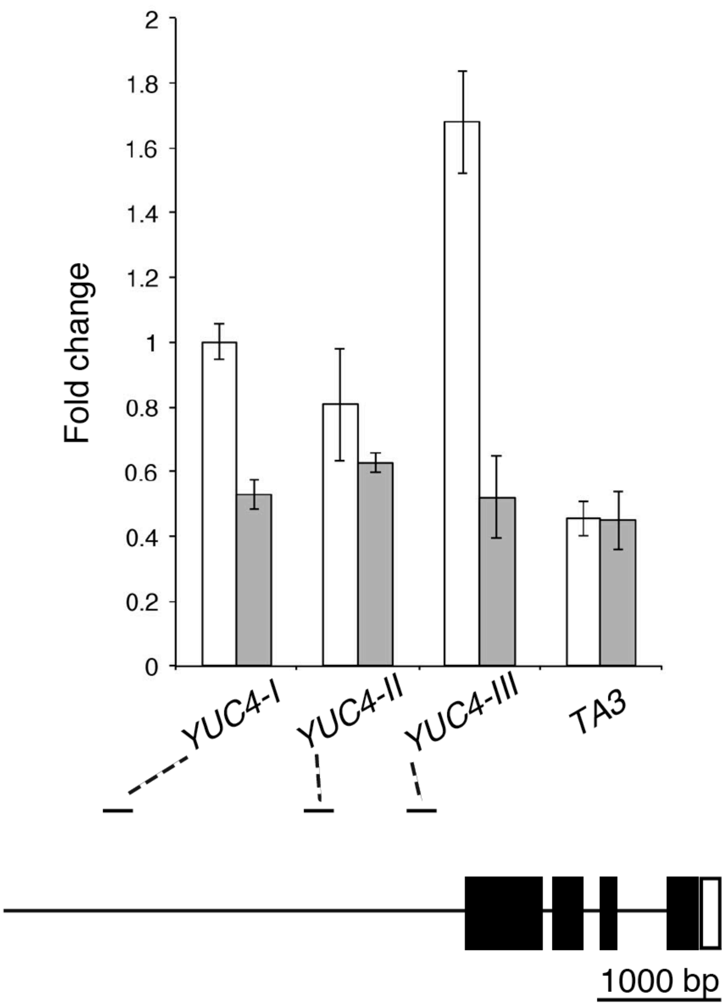

Supplementary Figure 14 AG ChIP enrichment at the *YUC4* locus. Top: Anti AG ChIP to test AG occupancy at the *YUC4* locus. The values are represented as the means ± SEMs. Bottom: Diagram of the ChIP–qPCR amplicons tested.

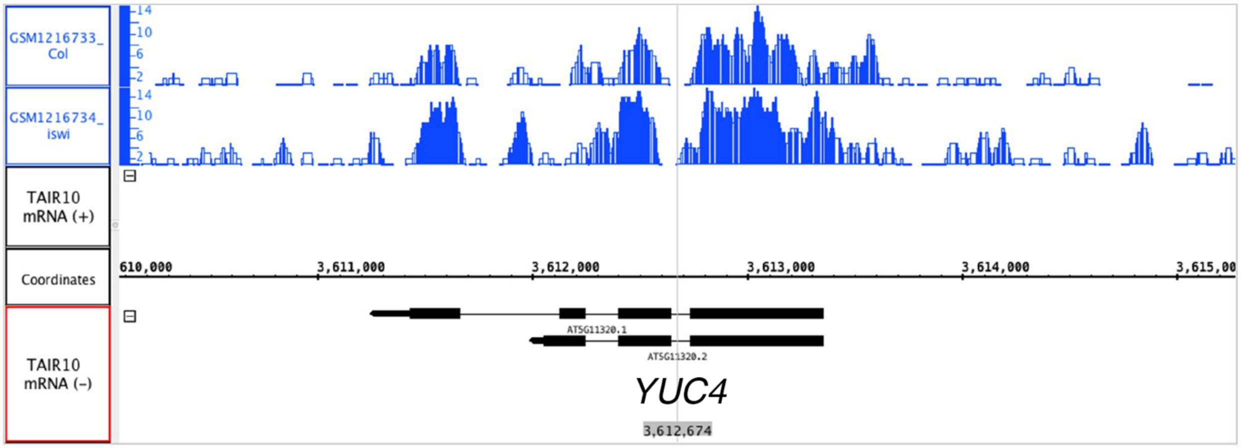

Supplementary Figure 15 Published MNase-seq data in wild-type and *chr11-1 chr17-1* plants. Nucleosome positioning identified by MNase-seq. Blue bars: Nucleosome occupancy. Black square: exon. Black line: intron.

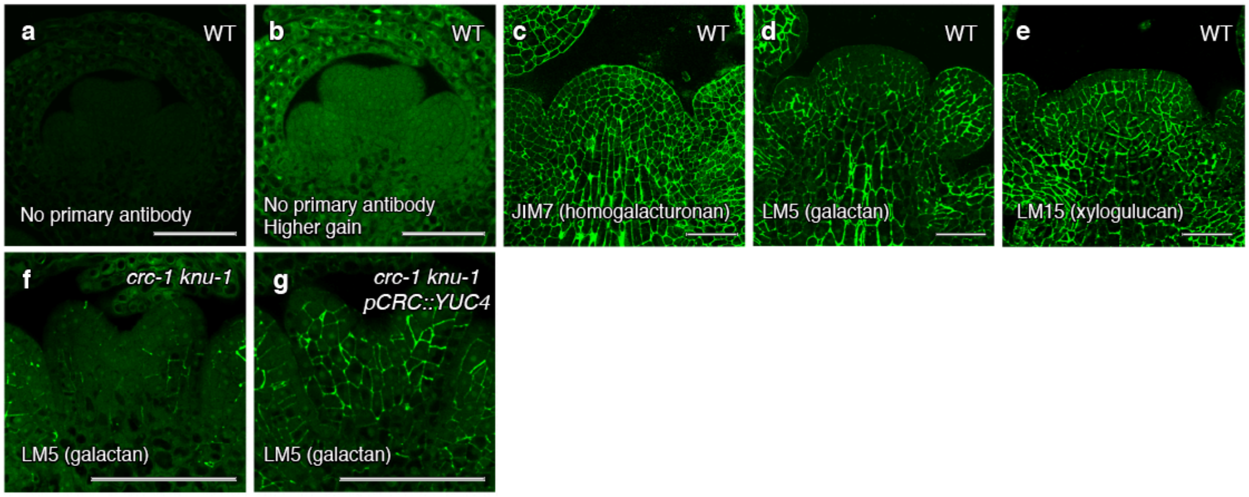

Supplementary Figure 16 Immunolocalization of cell wall components in the inflorescence meristem. **a**, **b** Immunolocalization using longitudinal sections of wild-type stage 6 flowers without the primary antibody. Without primary antibody (**a**). Without primary antibody when images were taken at higher gain settings. (**b**). Cell wall specific-fluorescence was not observed without primary antibody. The same section was used for **a** and **b**. **c** Immunolocalization of homogalacturonan epitopes was detected with JIM7 in longitudinal sections of the wild-type inflorescence meristem. **d** Immunolocalization of (1-4)-b-D-galactan epitopes was detected with LM5 in longitudinal sections of the wild-type inflorescence meristem. **e** Immunolocalization of xylogulucan epitopes was detected with LM15 using longitudinal sections of the wild-type inflorescence meristem. **f, g** Immunolocalization of (1-4)-b-D-galactan epitopes was detected with LM5 in longitudinal sections of the *crc-1 knu-1* (**f**) and *crc-1 knu-1 pCRC::YUC4* (**g**) terminating floral meristem. Half-strength of antibody was used. Bars = 50 μm in **a**-**g**.

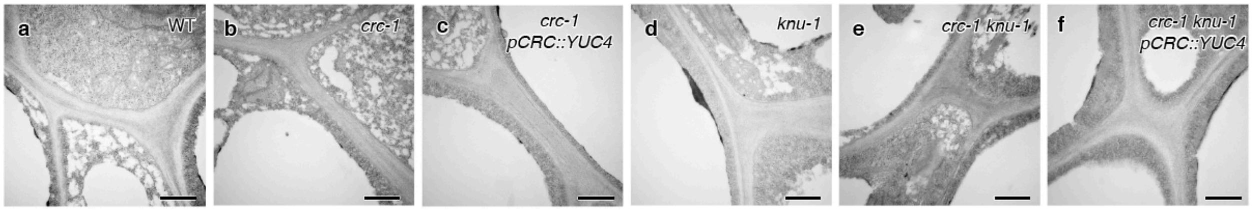

Supplementary Figure 17 TEM image of stage 6 terminating floral meristem cells. **a**–**f** TEM images of longitudinal sections from wild-type (**a**), *crc-1* (**b**), *crc-1 pCRC::YUC4* (**c**), *knu-1* (**d**), *crc-1 knu-1* (**e**), and *crc-1 knu-1 pCRC::YUC4* (**f**) terminating floral meristem at flower development stage 6. All images are shown at the same magnification. Bars = 500 nm in **a**–**f**.

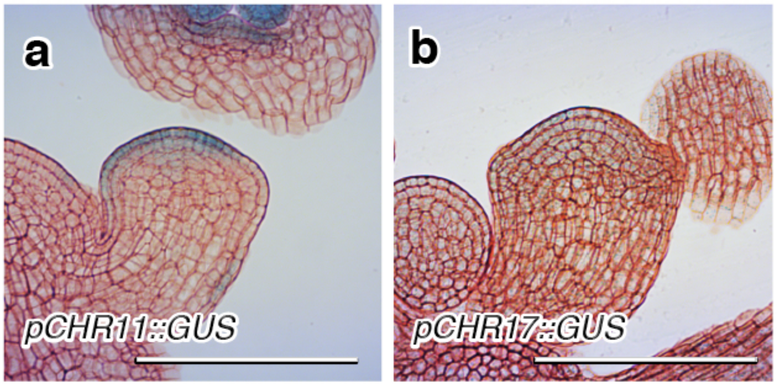

Supplementary Figure 18 Expression of *pCHR11::GUS* and *pCHR17::GUS* in stage 3 floral buds. **a**, **b** GUS staining of *pCHR11::GUS* (**a**) and *pCHR17::GUS* (**b**) in stage 3 floral meristems. Bars = 50 μm in **a**, **b**.

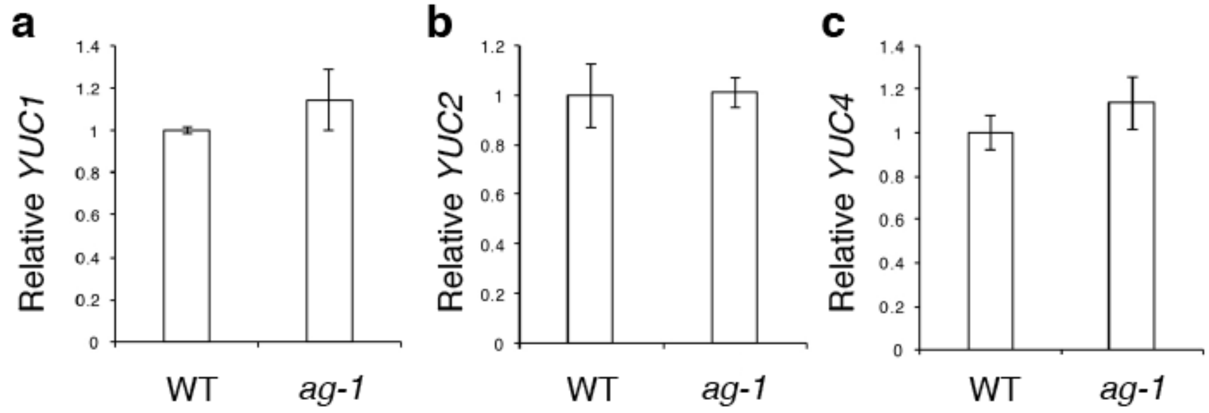

Supplementary Figure 19 Expression of *YUC* family genes in the wild type and *ag–1* mutant. **a–c** mRNA abundance of *YUC1* (**a**), *YUC2* (**b**), and *YUC6* (**c**) in wild–type and *ag–1* plants. The values are represented as the means ± SEMs.

Supplementary Table 1 Summary of transcriptomic datasets used to identify genes regulated by AG and CRC.

Dataset	Developmental stage/tissue	Number of genes	Annotation	Data References
WT vs *ag-12**	More than 3 weeks old (flowers at stage 12)	6673	TIGR 5.0	2
knu-1 vs *crc-1 knu-1**	Floral bud clusters up to stage 10	8150	TAIR 7	3
ag-11 ap2-35 vs *ag-11***	Floral bud clusters up to stage 7	2093	TAIR 10	4
ag-11 ap2-43 vs *ag-11***	Floral bud clusters up to stage 7	2026	TAIR 10	4
AG ChIP	Synchronized floral bud clusters at stage 5	1421	TAIR10	5

* Array data were downloaded from TAIR (ATGE_58A-C, ATGE_92A-C) and GEO (GSE88969) and reanalyzed. Genes showing at least a 1.5-fold change in expression were considered to be differentially expressed.
** The data were obtained from Huang et al., 2016[4]. Genes showing a 1.5-fold change in expression were considered to be differentially expressed, as written in Huang et al., 2016[4].

Supplementary References

1. H. Fukaki, Y. Nakao, Y. Okushima, A. Theologis, M. Tasaka, Tissue-specific expression of stabilized SOLITARY-ROOT/IAA14 alters lateral root development in Arabidopsis. *Plant J.* **44,** 382-395 (2005).

2. M. Schmid, T.S. Davison, S.R. Henz, U.J. Pape, M. Demar, M. Vingron, B. Scholkopf, D. Weigel, J.U. Lohman, A gene expression map of *Arabidopsis thaliana* development. *Nature Genet.* **37**, 501-506 (2005).

3. N. Yamaguchi, J. Huang, Y. Xu, K. Tanoi, T. Ito, Fine-tuning of auxin homeostasis governs the transition from floral stem cell maintenance to gynoecium formation. *Nature Communications* **8**, 1125 (2017).

4. Z. Huang, T. Shi, B. Zheng, R.E. Yumul, X. Liu, C. You, Z. Gao, L. Xiao, X. Chen, *APETALA2* antagonizes the transcriptional activity of *AGAMOUS* in regulating floral stem cells in *Arabidopsis thaliana*. *New Phytol.* **215**, 1197-1209 (2017).

5. D.S. OMaoileidigh, S.E. Wuest, L. Rae, A. Raganelli, P.T. Ryan, K. Kwasniewska, P. Das, A.J. Lohan, B. Loftus, E. Graciet, F. Wellmer, Control of reproductive floral organ identity specification in *Arabidopsis* by the C function regulator AGAMOUS. *Plant Cell* **25**, 2482-2503 (2013).
