# Supplementary material for: Chromatin-mediated feed-forward auxin biosynthesis in floral meristem determinacy
Source: Nat Commun. 2018 Dec 11;9:5290. doi: 10.1038/s41467-018-07763-0 (PMC6289996; doi:10.1038/s41467-018-07763-0)
Supplement: Supplementary file 2 — Description of Additional Supplementary Files [file 41467_2018_7763_MOESM2_ESM.docx]

**Title:** Supplementary Data 1
**Description:** Summary of the 53 genes downstream of AG and CRC.

**Title:** Supplementary Data 2
**Description:** GO term analysis of the 53 genes downstream of AG and CRC.

**Title:** Supplementary Data 3
**Description:** Primers used in this study.
