# Supplementary material for: Chromatin-mediated feed-forward auxin biosynthesis in floral meristem determinacy
Source: Nat Commun. 2018 Dec 11;9:5290. doi: 10.1038/s41467-018-07763-0 (PMC6289996; doi:10.1038/s41467-018-07763-0)
Supplement: Supplementary file 7 — Reporting Summary [file 41467_2018_7763_MOESM7_ESM.pdf]

nature research

Corresponding author(s): Toshiro Ito

Reporting Summary

Nature Research wishes to improve the reproducibility of the work that we publish. This form provides structure for consistency and transparency in reporting. For further information on Nature Research policies, see Authors & Referees and the Editorial Policy Checklist.

Statistical parameters

When statistical analyses are reported, confirm that the following items are present in the relevant location (e.g. figure legend, table legend, main text, or Methods section).

n/a	Confirmed	
☐	☒	The exact sample size (*n*) for each experimental group/condition, given as a discrete number and unit of measurement
☐	☒	An indication of whether measurements were taken from distinct samples or whether the same sample was measured repeatedly
☐	☒	The statistical test(s) used AND whether they are one- or two-sided<br>*Only common tests should be described solely by name; describe more complex techniques in the Methods section.*
☐	☒	A description of all covariates tested
☒	☐	A description of any assumptions or corrections, such as tests of normality and adjustment for multiple comparisons
☐	☒	A full description of the statistics including central tendency (e.g. means) or other basic estimates (e.g. regression coefficient) AND variation (e.g. standard deviation) or associated estimates of uncertainty (e.g. confidence intervals)
☒	☐	For null hypothesis testing, the test statistic (e.g. *F*, *t*, *r*) with confidence intervals, effect sizes, degrees of freedom and *P* value noted<br>*Give P values as exact values whenever suitable.*
☒	☐	For Bayesian analysis, information on the choice of priors and Markov chain Monte Carlo settings
☒	☐	For hierarchical and complex designs, identification of the appropriate level for tests and full reporting of outcomes
☒	☐	Estimates of effect sizes (e.g. Cohen's *d*, Pearson's *r*), indicating how they were calculated
☐	☒	Clearly defined error bars<br>*State explicitly what error bars represent (e.g. SD, SE, CI)*

Our web collection on statistics for biologists may be useful.

Software and code

Policy information about availability of computer code

Data collection	N/A
Data analysis	N/A

For manuscripts utilizing custom algorithms or software that are central to the research but not yet described in published literature, software must be made available to editors/reviewers upon request. We strongly encourage code deposition in a community repository (e.g. GitHub). See the Nature Research guidelines for submitting code & software for further information.

Data

Policy information about availability of data

All manuscripts must include a data availability statement. This statement should provide the following information, where applicable:

- Accession codes, unique identifiers, or web links for publicly available datasets
- A list of figures that have associated raw data
- A description of any restrictions on data availability

We only used publicly available datasets. ATGE_58A-C, ATGE_92A-C(https://www.arabidopsis.org). GSE88969, GSE81205(https://www.ncbi.nlm.nih.gov/geo/)

Field-specific reporting

Please select the best fit for your research. If you are not sure, read the appropriate sections before making your selection.

☒ Life sciences ☐ Behavioural & social sciences ☐ Ecological, evolutionary & environmental sciences

For a reference copy of the document with all sections, see nature.com/authors/policies/ReportingSummary-flat.pdf

Life sciences study design

All studies must disclose on these points even when the disclosure is negative.

Sample size	For statistical tests (i.e., Chi-Square, two-tailed Student's t-test), enough sample size was ensured (at least 30 plants for phenotyping, at least 3 biological replicates for expression or biochemical experiments) with more than 3 replicates each.
Data exclusions	Only experimental group and control group were included in the analyses. Any plants without these traits were excluded by genotyping.
Replication	At least three independent experiments were conducted for all experiments. Each experiment showed similar result.
Randomization	Random selection was not conducted. Plants in either experimental group or control group were included in the study. These two groups were distinct from each other; experimental group was compared with control group for phenotyping/expression.
Blinding	Blinding was not conducted. Plants in either experimental group or control group were included in the study. These two groups were distinct from each other; experimental group was compared with control group for phenotyping/expression.

Reporting for specific materials, systems and methods

Materials & experimental systems

n/a	Involved in the study	
☒	☐	Unique biological materials
☐	☒	Antibodies
☒	☐	Eukaryotic cell lines
☒	☐	Palaeontology
☒	☐	Animals and other organisms
☒	☐	Human research participants

Methods

n/a	Involved in the study	
☒	☐	ChIP-seq
☒	☐	Flow cytometry
☒	☐	MRI-based neuroimaging

Antibodies

Antibodies used	AG antibody; Ito et al., 1997. Myc antibody; Santa Cruz, 9E10. Pol II antibody; 4E8, Abcam. GFP antibody; A6455, Thermo Fisher Scientific. JIM7, LM5, LM15 antibody; Plant Probes.
Validation	AG antibody; Ito et al., 1997. Myc antibody; Ratsima H, et al., 2011, Proc Natl Acad Sci. 108: E914-23. Pol II antibody; Nguyen NH & Cheong JJ; Plant Physiol Biochem 130:14-19 (2018). GFP antibody; Valnegri et al., 2017, Nature Communications 8:1271. IM7, LM5, LM15 antibody; http://www.plantprobes.net/index.php
