# Supplementary material for: Chromatin-mediated feed-forward auxin biosynthesis in floral meristem determinacy
Source: Nat Commun. 2018 Dec 11;9:5290. doi: 10.1038/s41467-018-07763-0 (PMC6289996; doi:10.1038/s41467-018-07763-0)
Supplement: Supplementary file 6 — Peer Review File [file 41467_2018_7763_MOESM6_ESM.pdf]

Reviewers' Comments:

Reviewer #1:
Remarks to the Author:
The manuscript by Yamaguchi et al. describes the identification of common AG and CRC targets. One of them is YUCCA4, where the main focus is on, and the other target is TORNADO2, both affect auxin levels. The work described in the current manuscript is a continuation on their recently published work by Yamaguchi et al. (2017) in this same journal. The authors present a model, including chromatin modifications to explain a timing effect. The authors present that chromatin accessibility decreases in the chr11 chr 17 double mutant. The work gives more detailed understanding in floral meristem determination to allow gynoecium initiation. The authors perform bioinformatic analysis – using a lot of public data as well, followed by a lot of solid genetic experiments, as well as biochemistry. The paper is very well written, and all the data is clearly presented in nice figures. This paper will be a good addition to the current literature.

Some comments for the authors:
1) Did the authors check CRC and AG expression in the chr11 chr 17 double mutant? If they are reduced, it will also give reduced YUC4 expression.
2) It would have been nice to show YUC4::GUS expression in the chr11 chr 17 double mutant. The authors do show the reduction of YUC4 expression by qRT-PCR.
3) Can the carpel defects observed chr11 chr 17 be rescued by exogenous auxin application?
4) The immunolabelings are nice, though, the altered cell wall detection is difficult to see. The rescue of galactan (LM5 antibody) in the crc knu double mutant with YUC4 is difficult to see. It would be helpful if the authors could demonstrate this in a more quantitative manner, if not, maybe magnifications could help to observe better if there are differences.
5) Muller et al (2017) Plant Phys. reports on cytokinin-auxin crosstalk in the floral meristem-initiating gynoecium. They also found YUC4 in the floral meristem. The authors should cite and discuss the reported results.
6) Feed-forward loops between transcription factors have recently also been shown during early gynoecium development, also activating auxin biosynthesis, in Reyes-Olalde et al. (2017) PLoS Genetics. It would be nice to cite this paper as well.
7) It has been shown in the past by the Sundberg group that the crc phenotype can be rescued by auxin or NPA application. This work should be cited. The authors themselves also showed this in their recent paper by Yamaguchi et al (2017).
8) Why are three arrows red in the model in Fig. 7o?
9) Minor, in the reference list some reference miss the year of publication: refs 7, 37, 38, 69, 72.

Reviewer #2:
Remarks to the Author:
Chromatin-mediated feedforward auxin biosynthesis in floral meristem determinacy by Yamaguchi et al.

In this manuscript, the authors identified and characterized a common target of CRC and AG. They showed that AG and CRC synergistically activate the auxin biosynthesis gene YUC4. More importantly, they demonstrated that ectopic expression of YUC4 using the CRC promoter can partially suppress crc mutants. This is an interesting paper and the conclusions are largely supported by the data. The following comments may help the authors improve their manuscript.
1) The authors showed that CRM4 was an important cis-element for CRC-regulated YUC4 expression. They also stated that they did not detect any differences in the expression of other YUC genes between the wild
type and ag. Did the authors analyze the promoter regions of other YUCs? It would be interesting to delete the CRM4 element in the promoter by gene editing (they did the deletions in the GUS construct, but that is different).

2) The YUC genes are known to affect floral indeterminacy. For example, a multiple yuc mutant developed a gynoecium out of the top of the existing gynoecium (Cheng et al. 2006 Genes & Development). It would be very helpful if the authors can discuss their findings in the context of previous reports.

Reviewer #3:
Remarks to the Author:
This paper reveal additional information on floral meristem determinacy by providing, step by step, evidence on how AG and CRC execute their function in floral meristem determinacy. Performing data mining analysis, the authors identified the YUC4 gene as a direct target of AG and CRC, and by employing different methods, they demonstrate that AG and CRC binds to the YUC4 promoter and activate its expression. The authors showed a reduction in YUC4 expression in mutants of AG and CRC. They further demonstrate that induction of AG and CRC by dex led to upregulation of the YUC4 gene. By ChIP-qPCR they showed strong association of CRC-myc with a cis element in the YUC4 promoter and subsequently confirm the function of the cis element by mutated promoter-GUS analysis.
They further showed that the ectopic expression of YUC4 rescued the indeterminacy phenotype in crc knu which provides a strong evidence for the YUC4 capacity to contribute to the transition from floral stem cell maintenance to gynoecium formation.
All in all this is a comprehensive study that provides significant results and evidence for the direct regulation of YUC4 gene by AG and CRC and to the YUC4 contribution to the termination of floral meristem. This finding provides the link between CRC and regulation of auxin homeostasis.
The figures are clear, and the text reads well.

Though this is a high quality paper, my personal opinion is that it will not be interesting to scientists in other related disciplines and that it is fit better to specialist journals like Genes and Development. In a previous work from this lab Yamaguchi et al (Nat Commun. 2017) the authors identified a mechanistic link between floral meristem termination and gynoecium development through fine-tuning of auxin homeostasis by CRC. The current work revealing the potential link between CRC and auxin homeostasis, which deepen our understanding of how it is been executed, but does not report on a new mechanism, therefore it is less interesting to scientists in other related disciplines

Remarks:
1. The authors claim that in the wild type the YUC4 is expressed in "the abaxial carpels at stage 6 of flower development". I am not convinced with the GUS analysis presented and it is not clear why they chose the GUS approach over in situ, which is much more accurate.
I would add in the legend of figure 2 information on how many lines exhibited this pattern and whether the same line served in all mutant backgrounds.
2. The YUC4::GUS analysis shows that the gene is expressed in the sepal primordia. CRC and AG do not express in that domain and the authors suggest that in the sepal the expression is regulated by other factors. In light of the GUS analysis, the results of the qRT-PCR of YUC4 in Fig 2 (a-c) are surprising. How a reduction in a small domain (abaxial side of the carpel primordia) is not masked by the strong expression in the sepal?
The analysis was done on floral buds---(page 12 line 24) "Total RNA was extracted from Arabidopsis floral bud clusters up to stage 10".
It is important to refer to this issue in the text.

3. Fig 5 and 7: The panel of the SEM analysis doesn't contribute and it is redundant with the upper panel (a to f, look exactly the same like g to l). You present scanning electron microscope images for clos-ups that can provide additional information. This is not the case therefore I would take those images out.

Point-by-point Responses to referees' comments

Comments to reviewer #1

General comment by Reviewer #1

The manuscript by Yamaguchi et al. describes the identification of common AG and CRC targets. One of them is YUCCA4, where the main focus is on, and the other target is TORNADO2, both affect auxin levels. The work described in the current manuscript is a continuation on their recently published work by Yamaguchi et al. (2017) in this same journal. The authors present a model, including chromatin modifications to explain a timing effect. The authors present that chromatin accessibility decreases in the chr11 chr17 double mutant. The work gives more detailed understanding in floral meristem determination to allow gynoecium initiation. The authors perform bioinformatic analysis – using a lot of public data as well, followed by a lot of solid genetic experiments, as well as biochemistry. The paper is very well written, and all the data is clearly presented in nice figures. This paper will be a good addition to the current literature.

General Response

We are grateful to Reviewer 1 for the critical feedback, which has helped us improve our paper. We fully agree that we should characterize the *chr11 chr17* double mutant more carefully, since it displays pleiotropic phenotypes (Requests 1, 2, and 3). We conducted all three suggested experiments. 1) We have now included *CRC* and *AG* RT-PCR expression data to show specificity of gene expression changes in the *chr11 chr17* double mutant background. 2) Also, we have provided the *pYUC4::GUS* expression data to confirm that a reduction in spatial *YUC4* expression results in termination of the floral meristem in the *chr11 chr17* double mutant background. 3) Furthermore, we have treated the *chr11 chr17* double mutant with auxin. We believe that the revised version of our manuscript describes the role of CHR11 and CHR17 more precisely than the previous version. We hope that our manuscript will now be deemed suitable for publication. Please see our point-by-point responses below.

Request 1 by Reviewer 1

1) Did the authors check CRC and AG expression in the chr11 chr17 double

mutant? If they are reduced, it will also give reduced YUC4 expression.

Response 1

We have included the results of *AG* and *CRC* expression in the *chr11 chr17* double mutant by qRT-PCR analysis in Supplemental Fig. 13e and f. Also, we have included a discussion explaining why *CRC,* but not *AG,* was reduced in the *chr11 chr17* double mutant, in the revised version of our discussion. Briefly, *CRC* expression could be regulated by AG-CHR11/17, like *YUC4*. Furthermore, *CRC* reduction could also give reduced *YUC4* expression in the *chr11 chr17* double mutant background, as you pointed out.

Request 2 by Reviewer 1

2) It would have been nice to show YUC4::GUS expression in the chr11 chr 17 double mutant. The authors do show the reduction of YUC4 expression by qRT-PCR.

Response 2

We fully agree that we should have provided the results of the *YUC4::GUS* expression analysis in the wild type and *chr11 chr17* double mutant considering that the double mutants have pleiotropic phenotypes. In the revised version of our paper, we have included these data in the new Fig. 4d and e. Consistent with the qRT-PCR results, we observed reduced *YUC4* expression in *chr11 chr17* carpel primordia at stage 6 compared to the wild type.

Request 3 by Reviewer 1

3) Can the carpel defects observed chr11 chr17 be rescued by exogenous auxin application?

Response 3

To test whether a general increase in auxin levels within the gynoecium is sufficient to rescue the *chr11 chr17* phenotype, we treated developing *chr11 chr17* flowers with an optimal concentration of auxin. However, we did not observe phenotypic rescue. Perhaps, local accumulation of auxin is necessary for phenotypic rescue. We have included these data in Supplemental Fig. 13d.

Request 4 by Reviewer 1

4) The immunolabelings are nice, though, the altered cell wall detection is difficult to see. The rescue of galactan (LM5 antibody) in the crc knu double mutant with YUC4 is difficult to see. It would be helpful if the authors could demonstrate this in a more quantitative manner, if not, maybe magnifications could help to observe better if there are differences.

Response 4

As you pointed out, it would be better to quantify the amount of galactan by counting the number of gold particles binding per molecule of primary antibody using immunoelectron microscopy, as for example in Hongo et al., 2012 Plant Cell 26, 2624-2634. However, as it was technically challenging for us to perform such an analysis, we have replaced the *crc knu* figure into the new one. Also, we have included higher magnification images of the *crc knu* double mutants with and without *YUC4* in Supplemental Fig. 16, as you suggested. In the figure, we used half-strength of galactan antibody to observe quantitative differences more clearly.

Request 5 by Reviewer 1

5) Muller et al (2017) Plant Phys. reports on cytokinin-auxin crosstalk in the floral meristem-initiating gynoecium. They also found YUC4 in the floral meristem. The authors should cite and discuss the reported results.

Response 5

We agree that we should have cited the work by Muller et al. (2017) (47). In the revised version of our paper, we cited this paper and mentioned their results. Consistent with their findings, we observed *YUC4* expression in the apical and abaxial sides of carpels.

Request 6 by Reviewer 1

6) Feed-forward loops between transcription factors have recently also been shown during early gynoecium development, also activating auxin biosynthesis, in Reyes-Olalde et al. (2017) PLoS Genetics. It would be nice to cite this paper as well.

Response 6

Thank you for pointing out this important reference. Including the reference by

Reyes-Olalde et al. (2017) (63) would help us emphasize the general importance of feed-forward loops by transcription factors and auxin biosynthesis. Therefore, we cited this paper and mentioned it in our discussion. Further, our finding shows that this feedforward loop is mediated at the level of chromatin accessibility control.

Request 7 by Reviewer 1

7) It has been shown in the past by the Sundberg group that the crc phenotype can be rescued by auxin or NPA application. This work should be cited. The authors themselves also showed this in their recent paper by Yamaguchi et al (2017).

Response 7

We apologize for not citing the previous important paper by Staldal et al. (2008) (22), which initially showed rescue of the *crc* mutant by NPA application. We cited the paper in the revised version of our introduction.

Request 8 by Reviewer 1

8) Why are three arrows red in the model in Fig. 7o?

Response 8

The feed-forward loop we identified is shown in red. In the revised version of our paper, we explained that in the figure legend.

Request 9 by Reviewer 1

9) Minor, in the reference list some reference miss the year of publication: refs 7, 37, 38, 69, 72.

Response 9

We have added the year of publication accordingly. We also checked citations throughout the manuscript.

Comments to reviewer #2

General comment by Reviewer #2

Chromatin-mediated feedforward auxin biosynthesis in floral meristem determinacy by Yamaguchi et al. In this manuscript, the authors identified and characterized a common target of CRC and AG. They showed that AG and CRC synergistically activate the auxin biosynthesis gene YUC4. More importantly, they demonstrated that ectopic expression of YUC4 using the CRC promoter can partially suppress crc mutants. This is an interesting paper and the conclusions are largely supported by the data. The following comments may help the authors improve their manuscript.

General Response

We are grateful to Reviewer 2 for the constructive feedback. As indicated in the following responses, we have taken all of these comments and suggestions into account in the revised version of our manuscript. Specifically, we fully agree that we should clarify the role of *YUC4* among the *YUC* genes during floral meristem termination (Requests 1 and 2). 1) In the revised version of our paper, we compared the regulatory regions of the four key *YUC* genes, which play a key role during normal flower formation. Also, we constructed *YUC4* deletion lines by CRISPR/Cas to confirm the importance of its regulatory region. 2) We cited a previous key publication (Cheng et al., 2006), which describes the indeterminate phenotype seen in *yuc* multiple mutants, and discuss the findings of that study. Please see our point-by-point responses below.

Request 1 by Reviewer 2

1) The authors showed that CRM4 was an important cis-element for CRC-regulated YUC4 expression. They also stated that they did not detect any differences in the expression of other YUC genes between the wild type and ag. Did the authors analyze the promoter regions of other YUCs? It would be interesting to delete the CRM4 element in the promoter by gene editing (they did the deletions in the GUS construct, but that is different).

Response 1

We fully agree that we should characterize the similarities and differences of regulation of *YUC* family genes during flower development. 1) To examine the regulation of *YUC* genes, we compared their regulatory regions. Among 11 *YUC* family genes, *YUC4* and the three closest *YUC4* homologs (*YUC1*, *YUC2*, and

YUC6) play important roles during normal flower morphogenesis (Cheng et al., 2006). Only the *YUC4* regulatory region contains a flower-specific DNase I hypersensitive site containing potential YABBY binding sites (CRM4). We have included the results in Supplementary Fig. 10. 2). To further address the role of CRM4 in the *YUC4* promoter, we deleted this element by CRISPR-Cas9-mediated gene editing (Tsutsui et al., 2017 Plant Cell Physiol. (69)). We detected a reduction of *YUC4* mRNA in the deleted line. We have included these results in Supplementary Figure 9. These two pieces of evidence further support the importance of CRM4 in *YUC4*-mediated regulation in flowers.

Request 2 by Reviewer 2

2) The YUC genes are known to affect floral indeterminacy. For example, a multiple yuc mutant developed a gynoecium out of the top of the existing gynoecium (Cheng et al. 2006 Genes & Development). It would be very helpful if the authors can discuss their findings in the context of previous reports.

Response 2

Thank you for letting us know about this previous important finding. We think that including and discussing the reference by Cheng et al. (2006) (25) would help us emphasize the importance of *YUC* genes during floral meristem termination. According to this publication, an indeterminate phenotype was seen in the *yuc1 yuc2 yuc4* triple mutant, but not in either single mutant. Thus, we hypothesize that the effect of the *yuc4* mutation on floral indeterminacy is masked by the redundant activity of other *YUC* genes or other regulators in auxin pathways. Currently, the molecular mechanism by which the effect of *YUC4* is masked by other factors is unknown. However, one possible mechanism would involve a complex interaction between *YUC4* and *YUC* family member genes or other auxin-related factors (such as TRN2 and TAA1), since strong genetic interaction was often observed when *yuc* mutations were combined with other *yuc* mutants or auxin transport mutants (Cheng et al., 2006 (25) Gene dev, Cheng et al., 2007 Plant Cell). In the revised version of our paper, we have mentioned and discussed these previous results.

Comments to reviewer #3

General comment by Reviewer #3

This paper reveal additional information on floral meristem determinacy by providing, step by step, evidence on how AG and CRC execute their function in floral meristem determinacy. Performing data mining analysis, the authors identified the YUC4 gene as a direct target of AG and CRC, and by employing different methods, they demonstrate that AG and CRC binds to the YUC4 promoter and activate its expression. The authors showed a reduction in YUC4 expression in mutants of AG and CRC. They further demonstrate that induction of AG and CRC by dex led to upregulation of the YUC4 gene. By ChIP-qPCR they showed strong association of CRC-myc with a cis element in the YUC4 promoter and subsequently confirm the function of the cis element by mutated promoter-GUS analysis.

They further showed that the ectopic expression of YUC4 rescued the indeterminacy phenotype in crc knu which provides a strong evidence for the YUC4 capacity to contribute to the transition from floral stem cell maintenance to gynoecium formation.

All in all this is a comprehensive study that provides significant results and evidence for the direct regulation of YUC4 gene by AG and CRC and to the YUC4 contribution to the termination of floral meristem. This finding provides the link between CRC and regulation of auxin homeostasis. The figures are clear, and the text reads well.

Though this is a high quality paper, my personal opinion is that it will not be interesting to scientists in other related disciplines and that it is fit better to specialist journals like Genes and Development. In a previous work from this lab Yamaguchi et al (Nat Commun. 2017) the authors identified a mechanistic link between floral meristem termination and gynoecium development through fine-tuning of auxin homeostasis by CRC. The current work revealing the potential link between CRC and auxin homeostasis, which deepen our understanding of how it is been executed, but does not report on a new mechanism, therefore it is less interesting to scientists in other related disciplines.

General Response

We are grateful to Reviewer 3 for the critical feedback, which has helped us improve our paper. As indicated in the following responses, we have taken all of these comments and suggestions into account in the revised version of our manuscript. Specifically, we should have carefully designed and conducted gene expression analysis to examine the role of tissue-specific transcription factors in the previous version of our paper. 1) In the revised version of our paper, we have included *YUC4* in situ hybridization data to confirm the endogenous expression pattern and have described how we selected GUS lines. 2) We have used trimmed plants for our expression analysis to ascertain the difference between the wild type and mutants. We believe that the suggested experiments further support our original conclusion. Please see our point-by-point responses below.

Request 1 by Reviewer 3

1. The authors claim that in the wild type the YUC4 is expressed in "the abaxial carpels at stage 6 of flower development". I am not convinced with the GUS analysis presented and it is not clear why they chose the GUS approach over in situ, which is much more accurate. I would add in the legend of figure 2 information on how many lines exhibited this pattern and whether the same line served in all mutant backgrounds.

Response 1

We agree that we should confirm the endogenous expression pattern of *YUC4*. 1) We actually pre-screened the *pYUC4::GUS* line before we crossed the mutants with this line. Because signal strength was different between independent T1 lines, most likely due to positional effects of the transgene, we categorized the lines into three different groups. Regardless of signal strength, *YUC4* was expressed in the abaxial carpels at stage 6 of flower development. Since the majority (24 out of 37) of independent T1 lines showed strong *YUC4* expression, we used one representative line (line 6) for further crossing. We have provided the information in the legend of Supplementary Fig. 4a-c. Also, we mentioned that the same *pYUC4::GUS* line was used for expression analysis in the wild type and mutant background in the methods section. 2) We have included the results of our *YUC4* expression test by *in situ* hybridization in Supplementary Fig. 4d. Consistent with the *pYUC4::GUS* expression data,

stronger signal was detected from the abaxial carpels and sepals at stage 6 of flower development. 3) Our *pYUC4::GUS* and *YUC4* mRNA in situ data are largely in agreement with the previous report by Muller et al. (2017) (please also see our response to Request 5 by Reviewer 1). Since three independent experiments show similar data, we conclude that *YUC4* is expressed in the abaxial carpels at stage 6 of flower development.

Request 2 by Reviewer 3

2. The YUC4::GUS analysis shows that the gene is expressed in the sepal primordia. CRC and AG do not express in that domain and the authors suggest that in the sepal the expression is regulated by other factors. In light of the GUS analysis, the results of the qRT-PCR of YUC4 in Fig 2 (a-c) are surprising. How a reduction in a small domain (abaxial side of the carpel primordia) is not masked by the strong expression in the sepal?

The analysis was done on floral buds---(page 12 line 24) "Total RNA was extracted from Arabidopsis floral bud clusters up to stage 10".

It is important to refer to this issue in the text.

Response 2

We fully agree that we should have been careful about sample preparation. Although we detected a reduction in *YUC4* expression in the *ag* and *crc* mutants, this could be due to secondary effects, such as morphological changes (no and shorter carpels in *ag* and *crc* mutants, respectively. 1) To minimize tissue composition effects, we have newly repeated the RT-PCR analysis by using total RNA extracted from floral bud clusters up to stage 7. We also observed significant differences in the mutant background compared to controls. So, we have replaced these data (new Fig. 2a-c). Furthermore, we have mentioned stage information in the revised version of our text, as you suggested. Finally, we replaced the *YUC4* qRT-PCR data in the *chr11 chr17* double mutants as well, using RNA from floral bud clusters up to stage 7 (showed in new Fig 4c). 2) We tried to remove all floral buds older than stage 7; other tissues (such as sepals, pedicels etc.) were still included in the analysis. We believe that there are no differences in *YUC4* expression in the wild type, *ag*, and *crc* sepals, because *AG* and *CRC* are not expressed in sepals. Therefore, we explained why we

examined the spatial expression pattern in the revised version of our text.

Request 3 by Reviewer 3

3. Fig 5 and 7: The panel of the SEM analysis doesn't contribute and it is redundant with the upper panel (a to f, look exactly the same like g to l). You present scanning electron microscope images for clos-ups that can provide additional information. This is not the case therefore I would take those images out.

Response 3

We have modified the figures as you suggested.

Reviewers' Comments:

Reviewer #1:
Remarks to the Author:
The authors addressed all comments and included new experiments in the current manuscript. It is a nice story to be published.

Best,
Stefan de Folter.

Reviewer #2:
Remarks to the Author:
The authors have adequately addressed my concerns. However, the statement "Perhaps the local accumulation of auxin is important for phenotypic rescue." (line 17, page7) is not in the right tone. Local accumulation of auxin has been shown to be responsible for phenotypes in many mutants. For example, the yuc mutants were not rescued by exogenous auxin, but were rescued by YUCpro:iaaM.

Reviewer #3:
Remarks to the Author:
Dear Editor
I have carefully read the revised manuscript by Dr Ito and colleagues and thet Point-by-point Responses to referees' comments in the rebuttal document.
I am glad to say that the authors adequately addressed all the remarks I gave in my review
I think that the manuscript is of high quality meets suitable for publication in Nature communication

REVIEWERS' COMMENTS:

Comment by Reviewer #2:

The authors have adequately addressed my concerns. However, the statement "Perhaps the local accumulation of auxin is important for phenotypic rescue." (line 17, page7) is not in the right tone. Local accumulation of auxin has been shown to be responsible for phenotypes in many mutants. For example, the yuc mutants were not rescued by exogenous auxin, but were rescued by YUCpro:iaaM.

Response:

Thank you for pointing this out. In the revised version of our manuscript, we state "Local accumulation of auxin is important for phenotypic rescue in *chr11 chr17* as is often seen in many mutants [25]". Also, we cited the paper, which shows *yuc* mutant rescue not by exogenous auxin, but by *pYUC1::iaaM* (Cheng et al., 2006 Gene Dev.).
